# Antagonistic Interplay between Necdin and Bmi1 Controls Proliferation of Neural Precursor Cells in the Embryonic Mouse Neocortex

**DOI:** 10.1371/journal.pone.0084460

**Published:** 2014-01-02

**Authors:** Ryohei Minamide, Kazushiro Fujiwara, Koichi Hasegawa, Kazuaki Yoshikawa

**Affiliations:** Laboratory of Regulation of Neuronal Development, Institute for Protein Research, Osaka University, Suita, Osaka, Japan; Duke University Medical Center, United States of America

## Abstract

Neural precursor cells (NPCs) in the neocortex exhibit a high proliferation capacity during early embryonic development and give rise to cortical projection neurons after maturation. Necdin, a mammal-specific MAGE (melanoma antigen) family protein that possesses anti-mitotic and pro-survival activities, is expressed abundantly in postmitotic neurons and moderately in tissue-specific stem cells or progenitors. Necdin interacts with E2F transcription factors and suppresses E2F1-dependent transcriptional activation of the cyclin-dependent kinase *Cdk1* gene. Here we show that necdin serves as a suppressor of NPC proliferation in the embryonic neocortex. Necdin is moderately expressed in the ventricular zone of mouse embryonic neocortex, in which proliferative cell populations are significantly increased in necdin-null mice. In the neocortex of necdin-null embryos, expression of Cdk1 and Sox2, a stem cell marker, is significantly increased, whereas expression of p16, a cyclin-dependent kinase inhibitor, is markedly diminished. Cdk1 and p16 expression levels are also significantly increased and decreased, respectively, in primary NPCs prepared from necdin-null embryos. Intriguingly, necdin interacts directly with Bmi1, a Polycomb group protein that suppresses p16 expression and promotes NPC proliferation. In HEK293A cells transfected with luciferase reporter constructs, necdin relieves Bmi1-dependent repression of *p16* promoter activity, whereas Bmi1 counteracts necdin-mediated repression of E2F1-dependent *Cdk1* promoter activity. In lentivirus-infected primary NPCs, necdin overexpression increases *p16* expression, suppresses *Cdk1* expression, and inhibits NPC proliferation, whereas Bmi1 overexpression suppresses *p16* expression, increases *Cdk1* expression, and promotes NPC proliferation. Our data suggest that embryonic NPC proliferation in the neocortex is regulated by the antagonistic interplay between necdin and Bmi1.

## Introduction

Higher brain functions of mammals are performed by a vast number of neurons in the cerebral neocortex. A large population of neocortical neurons arises from NPCs or neural stem cells residing in the neural tube during early developmental period. Early NPCs proliferate by dividing symmetrically for self-renewal (expansion phase) and then asymmetrically to produce young neurons (neurogenic phase) [1]. Nascent neurons migrate radially to form the cortical plate, which gives rise to a typical six-layered structure of the neocortex after maturation. During early neocortical neurogenesis, NPCs proliferate rapidly to expand their pool because the number of postmitotic neurons correlates closely with that of NPCs. Although it is speculated that expansion of neocortical NPCs is tightly regulated in each mammalian species, there is limited information about molecular mechanisms underlying the regulation of neocortical NPC proliferation.

Cell cycle regulators are expressed in the neocortex at early stages of mammalian development [2], [3]. Regulation of the cell cycle is dependent on the control of cyclin-dependent kinases (Cdks), whose activities are positively regulated by cyclins and negatively by Cdk inhibitors such as p16^Ink4a^ (p16), p21^Cip1^ (p21) and p27^Kip1^ (p27). These inhibitors suppress Cdk activities and reduce phosphorylation of the retinoblastoma protein (Rb) family proteins such as Rb, p107, and p130. Hypophosphorylated Rb family proteins repress the activities of E2F family transcription factors that activate downstream genes involved in cell cycle progression. The Rb family proteins are differentially expressed in the embryonic brain during embryogenesis [2], [4]. However, detailed mechanisms whereby these cell cycle-related proteins regulate the self-renewal and proliferation of embryonic NPCs remain elusive.

Necdin was originally identified as a hypothetical protein encoded by a neural differentiation-induced gene in murine embryonal carcinoma P19 cells [5]. Necdin is abundantly expressed in virtually all of postmitotic neurons and skeletal muscle cells at early stages of development [6], [7]. Ectopic expression of necdin strongly suppresses the proliferation of tumor-derived cell lines [8]–[11]. Necdin, like Rb, binds to E2F1 and E2F4 [9], [12], and interacts with E2F1 on the *Cdk1* (*Cdc2*) promoter to repress the transcriptional activation of the *Cdk1* gene [13]. Thus, necdin is likely to downregulate the expression of E2F-dependent cell cycle-related genes in proliferative cells and exerts its anti-mitotic activity during neurogenesis. However, there is little information about the molecular mechanism whereby necdin controls cell divisions of NPCs during embryonic neurogenesis.

Accumulating evidence has demonstrated that necdin is moderately expressed in tissue-specific stem cells or progenitors such as mesoangioblast stem cells [14], brown adipocyte precursors [15], skeletal muscle satellite cells [16], hematopoietic stem cells [17]–[19], white adipocyte progenitor cells [20], and NPCs in the ganglionic eminences (GEs) [21]. It has been suggested that necdin regulates the proliferation and quiescence of several tissue-specific stem cells and progenitors [17]–[21]. These previous findings prompted us to investigate whether necdin controls NPC proliferation in the embryonic neocortex.

In this study, we examined whether necdin regulates proliferation of neocortical NPCs in necdin-null mouse embryos. Analyses using neocortical NPCs prepared from necdin-null mice show that necdin suppresses NPC proliferation and induces significant changes in p16 and Cdk1 expression. We also demonstrate that necdin and Bmi1, a key transcription factor involved in NPC proliferation, interact to modulate their downstream cell-cycle regulatory systems. The present study provides insights into the regulatory mechanisms underlying NPC proliferation in the mammalian neocortex.

## Results

### Necdin Deficiency Increases Proliferative Cell Populations in the Embryonic Neocortex *in vivo*

**Figure 1:**
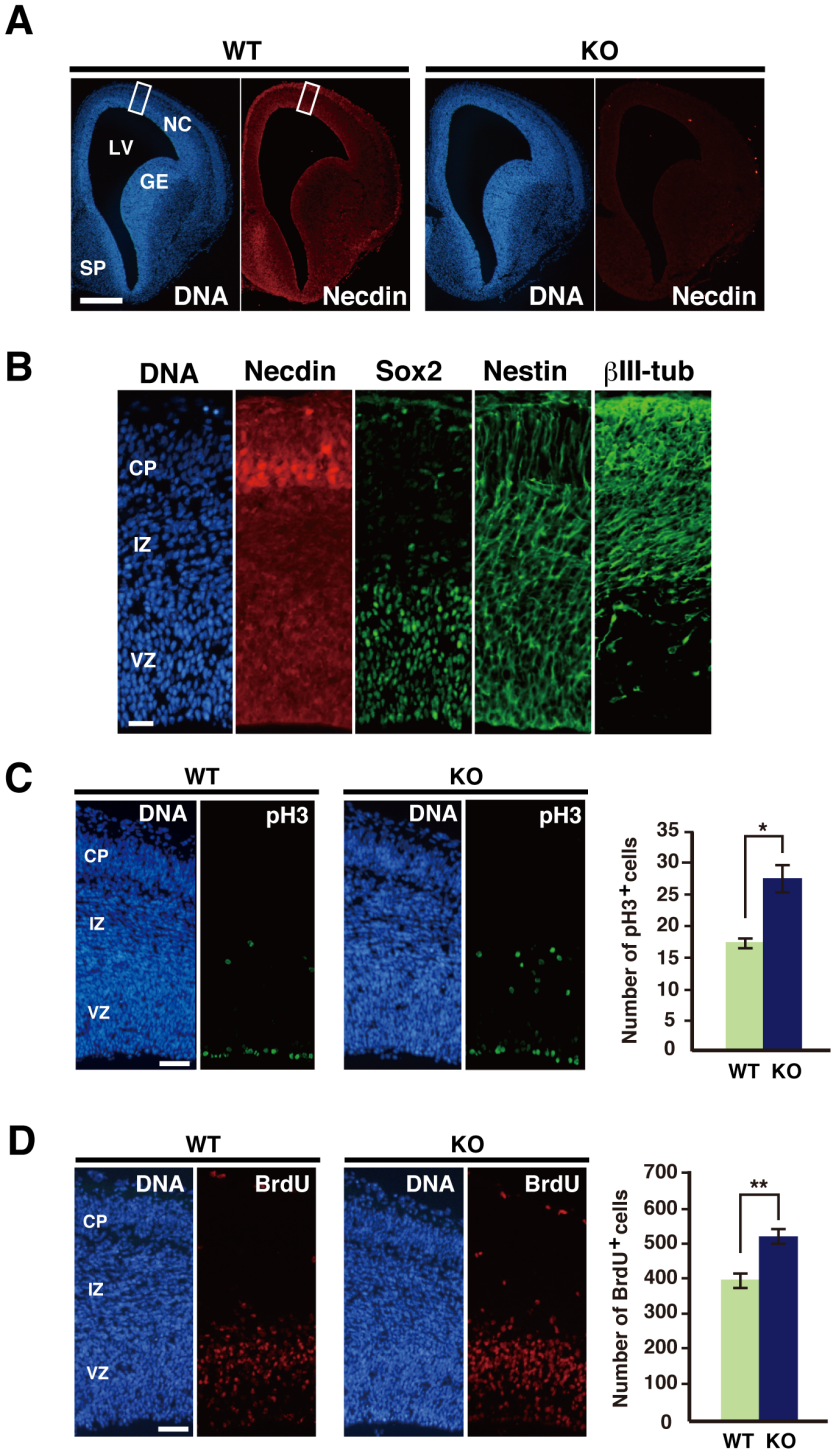
Necdin deficiency increases proliferative cell populations in the embryonic neocortex. (**A**) Distribution of necdin in the forebrain. Cryosections were prepared from wild-type (WT) and necdin-null (KO) mice at E14.5 and immunostained for necdin. Abbreviations: NC, neocortex; GE, ganglionic eminence; LV, lateral ventricle; SP, septum. (**B**) Distribution of necdin, Sox2, nestin, and βIII-tubulin (βIII-tub) in the neocortex. The area shown is boxed in (**A**). Abbreviations: CP, cortical plate; IZ, intermediate zone; VZ, ventricular zone. (**C**) Phospho-histone H3 (pH3) immunohistochemistry. Cryosections were prepared from wild-type (WT) and necdin-null (KO) mice at E14.5, and immunostained for pH3. (**D**) S-phase cell population. Forebrain sections of E14.5 embryos were prepared 4 hrs after BrdU injection into pregnant female mice. BrdU^+^ cells in the neocortex were detected by immunohistochemistry. Chromosomal DNA (DNA) was stained with Hoechst 33342 in (**A–D**). pH3^+^ and BrdU^+^ cells within each 200-µm-wide radial column of the neocortex are counted (mean ± SEM, *n = *3). *p<0.05, **p<0.01. Scale bars, 250 µm in (**A**), 100 µm in (**B**), 50 µm in (**C**, **D**).

We examined the distribution pattern of necdin in the embryonic forebrain at E14.5 by immunohistochemistry (Fig. 1A). The necdin immunoreactivity was detected mainly in the cerebral neocortex and septum, consistent with our previous data [22]. In contrast, little necdin immunoreactivity was detected in the forebrain of necdin-null mice. In the neocortex, necdin was strongly expressed in the cortical plate (CP) where βIII-tubulin-positive (βIII-tubulin^+^) neurons were located (Fig. 1B). Necdin was also expressed moderately in the proliferative zone including the ventricular zone (VZ) where the stem cell markers Sox2 and nestin were expressed.

Because necdin has an anti-mitotic activity, we tested whether proliferative cell populations are increased in the neocortex of necdin-null mice at E14.5. The population expressing the mitosis marker phospho-histone H3 (pH3) increased by 62% in necdin-null neocortex (Fig. 1C). The numbers of pH3^+^ cells were increased in the apical and basal regions of the proliferative zone by 61% and 67%, respectively, of control levels. To determine whether the increased proliferative cell populations in necdin-null neocortex are limited to specific developmental periods, we analyzed the pH3^+^ cell population in the necdin-null neocortex at E12.5 and E16.5 (Fig. S1). The numbers of pH3^+^ neocortical cells in necdin-null mice significantly increased at E12.5 and E16.5 by 65% and 27%, respectively, suggesting that necdin negatively regulates the proliferation of NPCs throughout the period of embryonic neurogenesis. When the number of DNA-synthesizing cells in the neocortex of E14.5 mice was analyzed by 5′-bromo-2′-deoxyuridine (BrdU) incorporation assay, the BrdU^+^ cell population significantly increased by 28% in necdin-null neocortex (Fig. 1D). These changes of proliferative cell populations were observed predominantly in the proliferative zone of necdin-null mice.

Although we found significant increases in proliferative cell populations in necdin-null neocortex *in vivo*, there was little or no difference in the cortical thickness at E14.5 (Fig. S2). We assumed that the balance between proliferation and death of NPCs is maintained, resulting in no apparent change of the neocortical thickness. We thus analyzed apoptosis in the neocortex of E14.5 mice by terminal deoxynucleotidyl transferase-mediated dUTP nick end labeling (TUNEL). The number of TUNEL^+^ cells significantly increased in the proliferative zone of necdin-null mice at E12.5, E14.5, and E16.5 (Fig. S3), suggesting that both proliferation and apoptosis of NPCs are enhanced in developing neocortex of necdin-null mice.

We also examined whether necdin deficiency affects the number of neurons in the postnatal period, when the six-layered neocortical structure becomes evident (Fig. S4). The population of E14.5-born neurons, which were labeled with the thymidine analog 5′-ethynyl-2′-deoxyuridine (EdU), significantly increased at postnatal day 4 (P4) in necdin-null mice (WT, 143±6, KO, 166±8; *n = *3; *p*<0.01). In necdin-null mice, the number of E14.5-born neurons with weak EdU signals increased significantly (WT, 54±2, KO, 99±4; *n = *3; *p*<0.01), and these neurons were accumulated in the superficial layers of the neocortex (layers II/III), suggesting that necdin-null NPCs undergo subsequent cell divisions prior to terminal mitosis.

### Necdin Deficiency Affects Expression Levels of Cell Cycle-related Genes in the Embryonic Neocortex

**Figure 2:**
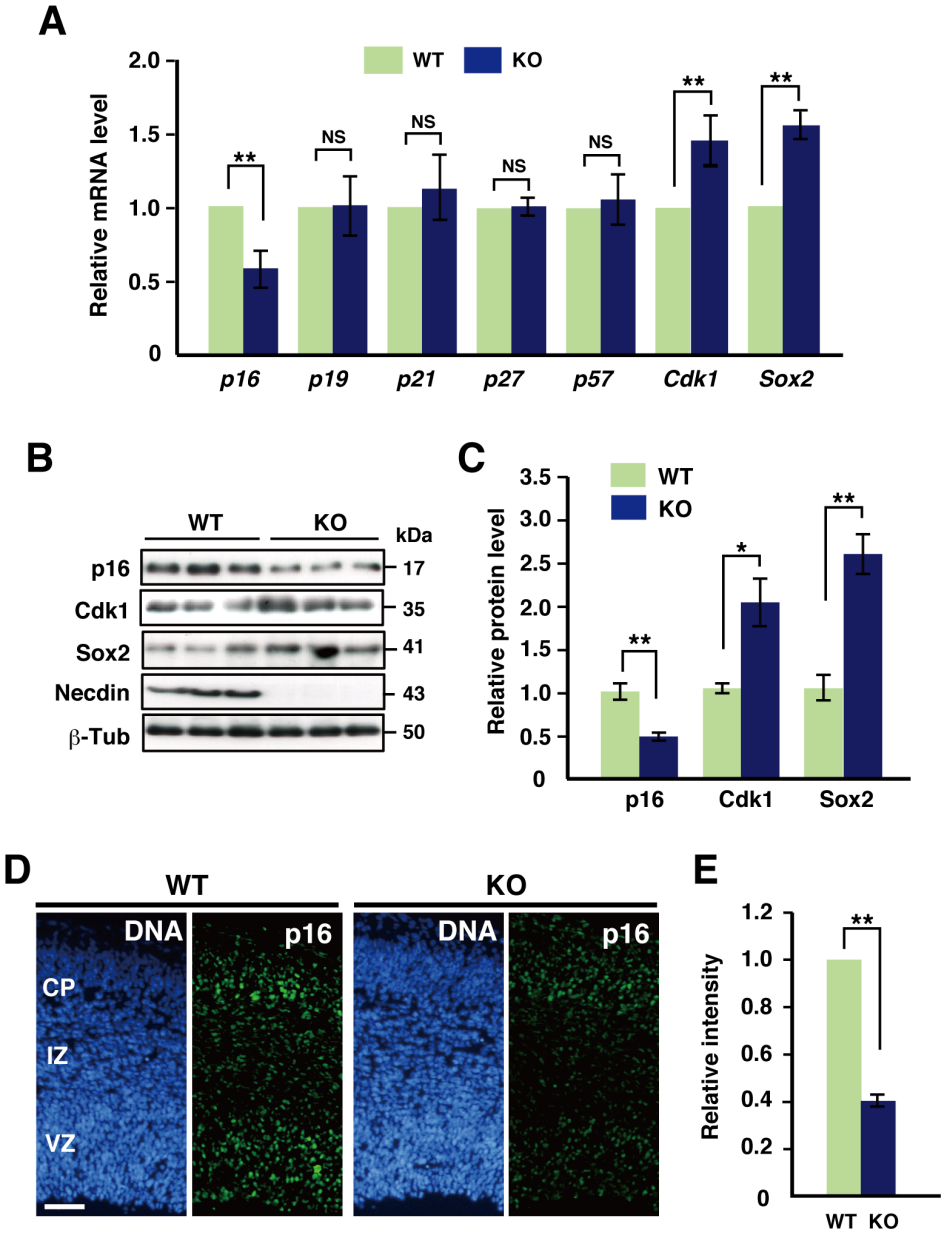
Necdin deficiency decreases *p16* expression and increases *Cdk1* expression in the embryonic neocortex. (**A**) mRNA levels of cell cycle regulators. Expression levels of the cell cycle-regulatory genes *p16*, *p19*, *p21*, *p27*, *p57*, *Cdk1* and the stem cell marker gene *Sox2* in the neocortex of wild-type (WT) and necdin-null (KO) mice at E14.5 were analyzed by qRT-PCR. (**B**, **C**) p16 and Cdk1 protein levels. Protein levels of p16, Cdk1, Sox2, necdin and β-tubulin (β-Tub) in the neocortex were analyzed by Western blotting and quantified by densitometry (**C**). Protein levels were normalized to β-tubulin levels. (**D**) Distribution of p16^+^ cells in the neocortex. Forebrain cryosections of E14.5 mice were immunostained for p16 and quantified by fluorescence microphotometry (**E**). Fluorescence intensity in each 200-µm-wide column was analyzed (mean ± SEM, *n = *3), *p<0.05, **p<0.01, NS, not significant. Scale bar, 50 µm.

To elucidate the molecular mechanism that increases NPC proliferation in necdin-null mice, we determined the mRNA levels of cell cycle regulators in E14.5 mouse neocortex *in vivo* by quantitative reverse-transcription PCR (qRT-PCR) (Fig. 2A). The *p16* mRNA level was significantly low in the necdin-null neocortex, whereas no significant differences in the *p19*, *p21*, *p27*, and *p57* mRNA levels were noted between wild-type and necdin-null mice. In contrast, the expression levels of *Cdk1* and *Sox2* mRNAs were significantly high in the neocortex of necdin-null mice. To examine whether these mRNA levels correlate with the protein levels, we analyzed the protein levels of p16, Cdk1, and Sox2 by Western blotting (Fig. 2B, C). In the neocortex of necdin-null mice, the p16 protein level decreased to 45% of the control level, whereas the Cdk1 and Sox2 protein levels increased 2.0- and 2.6-fold, respectively. We then examined the distribution pattern of p16^+^ cells in the neocortex of E14.5 mice by immunohistochemistry (Fig. 2D, E). The p16 protein was mainly detected in the CP and VZ. Fluorescence microphotometry revealed that the p16 protein level in the necdin-null neocortex markedly decreased to 40% of the wild-type control level. These results suggest that necdin deficiency downregulates p16 expression and upregulates Cdk1 expression in the neocortex to increase the NPC population *in vivo*.

### Necdin Deficiency Enhances Proliferation of Neocortical NPCs *in vitro*

**Figure 3:**
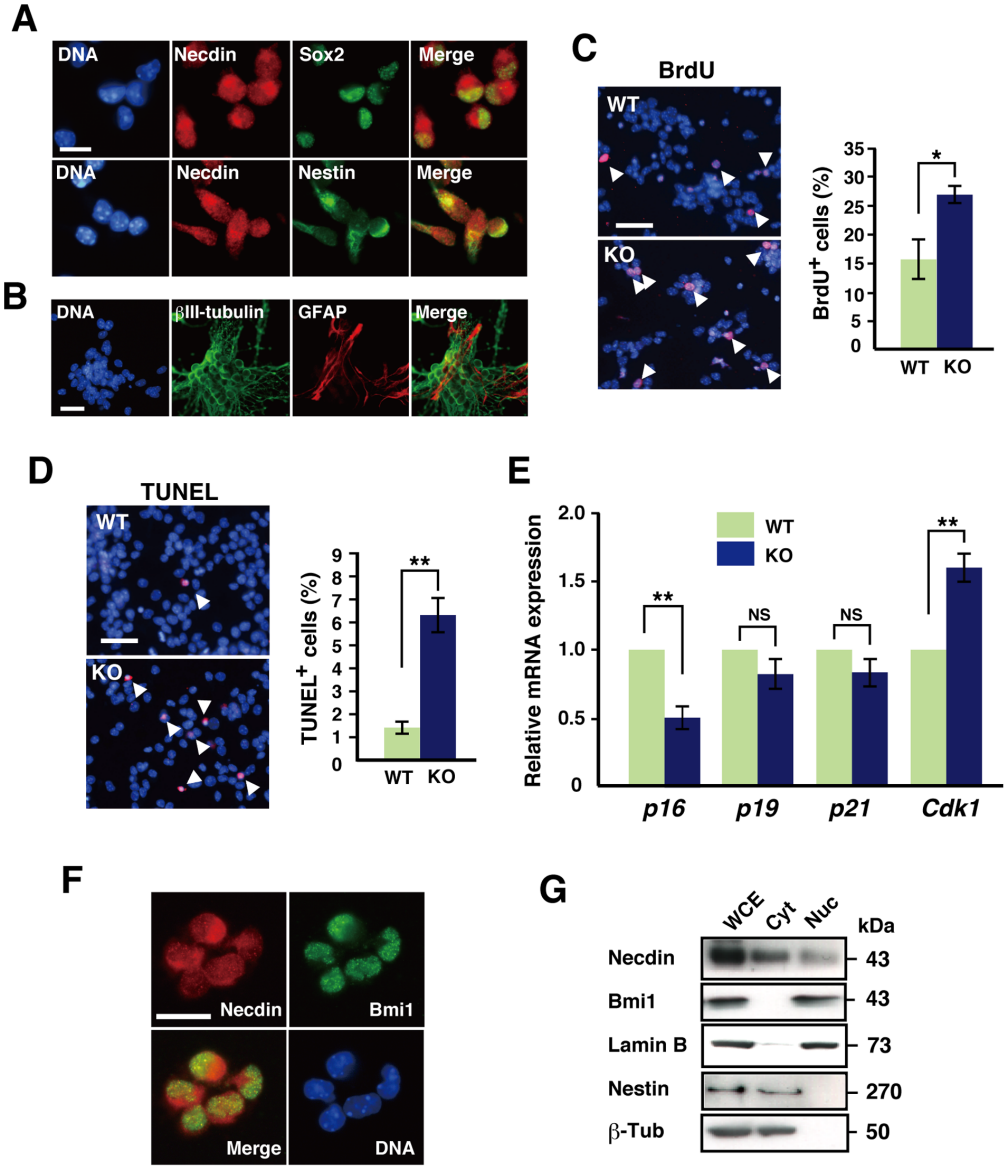
Necdin deficiency decreases *p16* expression and increases *Cdk1* expression in primary NPCs. (**A**) Expression of necdin, Sox2, and nestin in primary NPCs. Primary NPCs were prepared from the neocortex at E14.5 and subjected to double-immunostaining for necdin and Sox2 or nestin. (**B**) Double-staining for βIII-tubulin (green) and GFAP (red) in differentiated cells. Primary NPCs were induced to differentiate by growth factor withdrawal. (**C**, **D**) BrdU incorporation and TUNEL analyses. Primary NPCs were immunostained for BrdU (**C**) or labeled by TUNEL (**D**), and BrdU^+^ or TUNEL^+^ cells (red) were counted (total 100 cells examined). Arrows point to representative positive cells. Chromosomal DNA (DNA) was stained with Hoechst 33342 (blue) (**A**−**D**). (**E**) Expression of cell cycle-regulatory genes. *p16*, *p19*, *p21*, and *Cdk1* mRNAs expressed in NPCs were analyzed by qRT-PCR. (**F**) Coexpression of necdin and Bmi1 in NPCs. Primary NPCs were double-stained for necdin and Bmi1. (**G**) Subcellular distribution of necdin and Bmi1. The nucleus and cytoplasm of NPCs were fractionated, and the protein levels were analyzed by Western blotting. Abbreviations: WCE, whole cell extract; Cyt, cytoplasm; Nuc, nucleus. Lamin B was used as a nuclear marker, and nestin and β-tubulin (β-Tub) as cytoplasmic markers. Values in (**C**−**E**) represent the mean ± SEM (*n = *3), *p<0.05, **p<0.01, NS, not significant. Scale bars; 20 µm in (**A**, **B, F**), 50 µm in (**C, D**).

To examine whether necdin is expressed in neocortical NPCs, we prepared NPCs from the neocortex at E14.5, cultured for 48 hrs in the presence of epidermal growth factor (EGF) and basic fibroblast growth factor (bFGF), and carried out immunocytochemical analysis (Fig. 3A). Necdin was expressed in virtually all NPCs, most of which also expressed Sox2 and nestin (necdin^+^/Sox2^+^, 90±2%; necdin^+^/nestin^+^, 91±3%; total 200 cells analyzed; *n* = 3). The necdin immunoreactivity was detected in the cytoplasm and nucleus of these NPCs. When neocortical NPCs were differentiated *in vitro* in the absence of EGF and bFGF, βIII-tubulin^+^ neurons and glial fibrillary acidic protein (GFAP)^+^ astrocyte-like cells were differentiated, suggesting that primary NPCs are multipotent (Fig. 3B).

To determine whether necdin regulates the proliferation of NPCs in a cell-intrinsic manner, we analyzed BrdU incorporation into nuclear DNA of primary NPCs. In necdin-null NPCs, the number of BrdU^+^ cells was 1.69 times that in wild-type NPCs (Fig. 3C). In addition, the TUNEL^+^ cell population in necdin-null NPCs was 4 times that in wild-type NPCs (Fig. 3D). These results suggest that necdin suppresses both proliferation and apoptosis of primary NPCs as seen in the neocortex *in vivo*. We also analyzed the expression levels of cell cycle-regulatory genes in NPCs by qRT-PCR (Fig. 3E). In necdin-null NPCs, the *p16* mRNA expression level was 50% of that in wild-type NPCs, whereas the *Cdk1* mRNA level was 1.6 times the control level. In contrast, the expression levels of *p19* and *p21* mRNAs were unchanged.

Because p16 expression was markedly diminished in necdin-null NPCs, we investigated whether necdin increases p16 expression by suppressing Bmi1, a Polycomb group protein that represses p16 expression to promote NPC proliferation [23]. We examined the subcellular localization of necdin and Bmi1 in primary NPCs by immunocytochemistry (Fig. 3F) and Western blotting (Fig. 3G). Necdin was mainly present in the cytoplasm but moderately in the nucleus of primary NPCs, whereas Bmi1 was restricted to the nucleus. We then analyzed the expression levels of necdin and Bmi1 mRNAs in NPCs and neurons by qRT-PCR. The necdin mRNA level in differentiated neurons was 2.5 times the NPC level (Fig. S5). In contrast, the *Bmi1* mRNA level in NPCs was 3.1 times that in differentiated neurons. There was no difference in the *Bmi1* expression level between wild-type and necdin-null mice in neocortical NPCs or neurons. These results suggest that necdin and Bmi1 are coexpressed in the nucleus of neocortical NPCs, in which the *Bmi1* mRNA level is regulated in a necdin-independent manner.

### Necdin Physically Interacts with Bmi1

**Figure 4:**
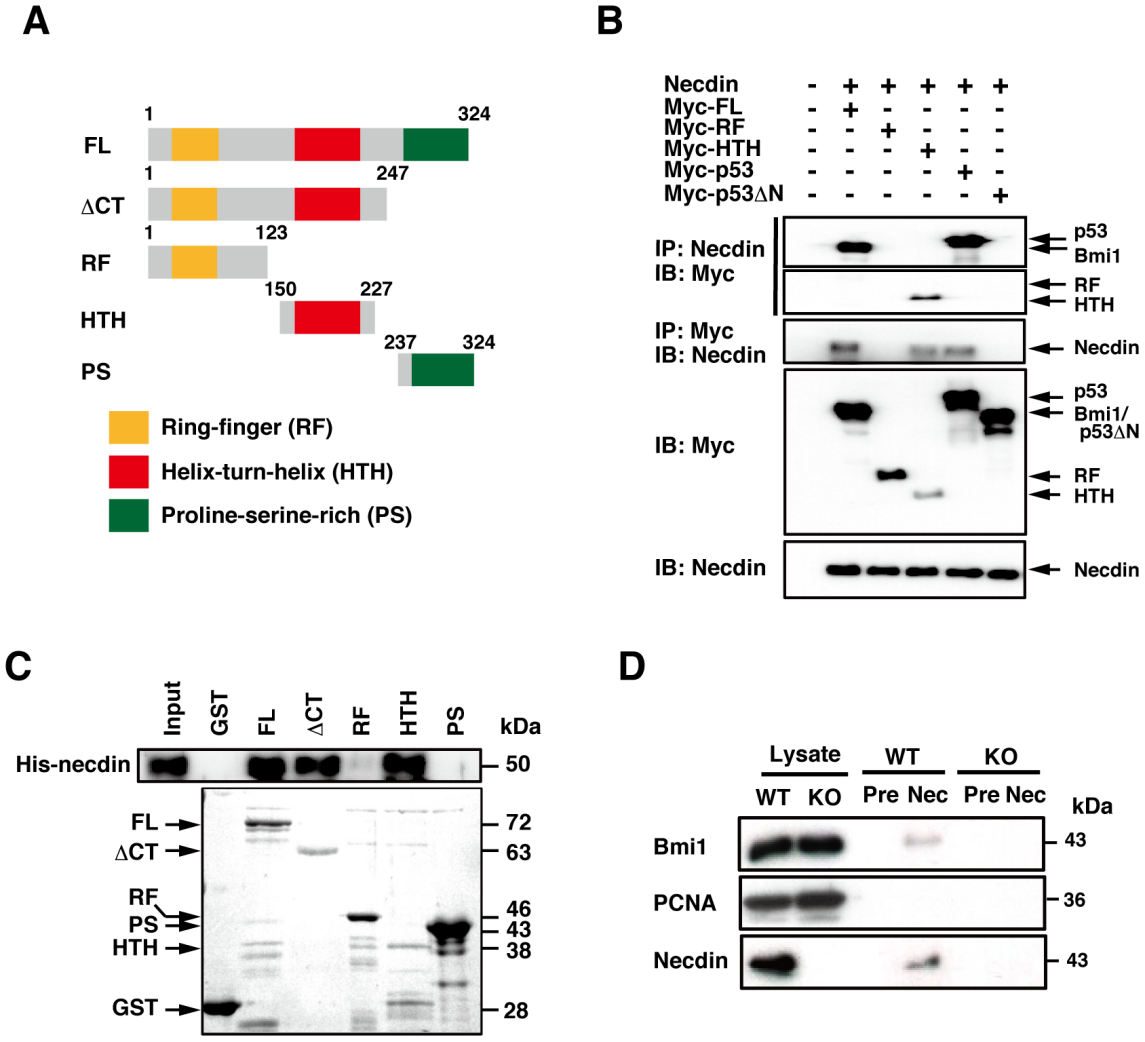
Necdin interacts with Bmi1 *in vivo* and *in vitro*. (**A**) Bmi1 deletion mutants. Bmi1 full-length (FL), C-terminal deletion (ΔCT), and mutants containing the Ring finger (RF), helix-turn-helix (HTH) and proline/serine-rich (PS) domains are schematically shown. (**B**) Co-immunoprecipitation assay. HEK293A cells were transfected with expression vectors for necdin and Myc-tagged FL, RF, HTH, p53 (positive control), and p53ΔN (negative control). Cell lysates were immunoprecipitated (IP) and immunoblotted (IB) with anti-Myc (Myc) and anti-necdin (Necdin) antibodies. (**C**) *In vitro* binding assay. GST-Bmi1 mutants immobilized on glutathione-agarose were incubated with His-tagged necdin (His-necdin), and bound His-necdin was detected by immunoblotting with anti-necdin antibody (upper panel). GST-Bmi1 deletion mutants were stained with Coomassie Brilliant Blue (lower panel). Arrows indicate the predicted protein positions (**B**, **C**). (**D**) Co-immunoprecipitation assay for endogenous complex containing necdin and Bmi1 in primary NPCs. Lysates of NPCs prepared from E14.5 wild-type (WT) and necdin-null (KO) mice were immunoprecipitated with anti-necdin IgG (Nec) or control preimmune IgG (Pre). Bmi1, PCNA (negative control), and necdin were detected by Western blotting. Lysate, tissue lysate (10 µg).

To determine whether necdin binds to Bmi1 by the protein-protein interaction, we constructed deletion mutants of Bmi1 encompassing known motifs such as the Ring-finger (RF), helix-turn-helix (HTH), and proline-serine-rich domains (PS) [24], [25] (Fig. 4A). When necdin and Myc-tagged Bmi1 deletion mutants (ΔCT, RF, HTH) were coexpressed in HEK293A cells, Bmi1 full-length (FL) and HTH were co-precipitated with necdin, and necdin was conversely co-precipitated with FL and HTH (Fig. 4B). The positive control p53, but not p53ΔN lacking the N-terminal necdin-binding site [26], was co-precipitated with necdin. In this assay, the HTH expression level was low, and expression of the C-terminal proline-serine-rich (PS) mutant was undetectable (not shown), presumably owing to the instability of these mutant proteins [27]. We next examined the direct interactions between necdin and Bmi1 deletion mutants by *in vitro* binding assay using bacterially expressed GST-tagged Bmi1 mutants and virally expressed His-tagged necdin (Fig. 4C). Necdin bound efficiently to Bmi1 FL, C-terminal deletion mutant (ΔCT) and HTH, but failed to bind to the N-terminal RF or C-terminal PS. These results suggest that necdin directly binds to Bmi1 via the central HTH domain. To detect an endogenous complex of necdin and Bmi1, we carried out the co-immunoprecipitation assay using neocortical NPC lysates prepared from wild-type and necdin-null mice at E14.5 (Fig. 4D). Bmi1 was co-precipitated with necdin in the NPC extract prepared from wild-type mice, but not with proliferating cell nuclear antigen (PCNA) used as a negative control, suggesting that Bmi1 and necdin interact endogenously in primary NPCs.

### Necdin and Bmi1 Counteract each other to Control Cdk1 and p16 Promoter Activities

**Figure 5:**
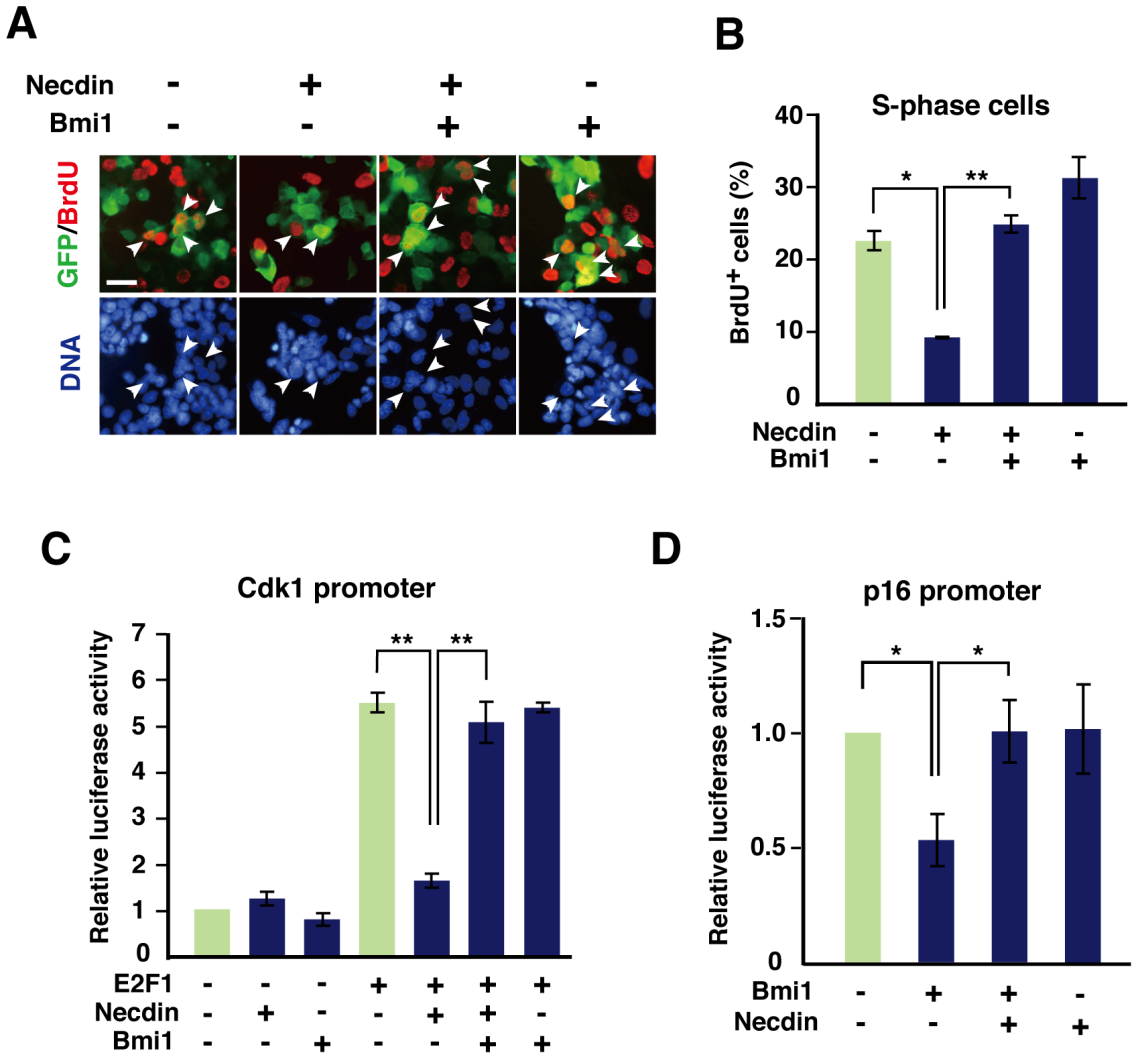
Necdin and Bmi1 relieve transcriptional suppression of the *p16* and *Cdk1* promoters. (**A**, **B**) BrdU incorporation assay. HEK293A cells transfected with expression vectors for GFP, necdin, and Bmi1 were immunostained for GFP (green) and BrdU (red) (**A**). BrdU^+^ cells among 500 GFP-expressing cells were counted (*n = *3) (**B**). Arrowheads point to GFP^+^/BrdU^+^ cells. Scale bar, 10 µm. (**C**, **D**): Promoter assay. Luciferase reporter vectors containing the *Cdk1* (**C**) and *p16* (**D**) promoters and expression vectors for E2F1, necdin, and Myc-tagged full-length Bmi1 (Bmi1) were co-transfected into HEK293A cells. Promoter activities were measured with a luminometer. Values in (**B**−**D**) represent the mean ± SEM, *n = *3. *p<0.05, **p<0.01.

We investigated whether the interaction between necdin and Bmi1 affects the proliferation rate of HEK293A by BrdU incorporation assay (Fig. 5A). Necdin reduced the BrdU^+^ cell population by 59% (Fig. 5B). Bmi1 efficiently counteracted the proliferation-suppressive activity of necdin. We then performed the luciferase reporter assay for *Cdk1* promoter activity (Fig. 5C). Necdin had no appreciable effect on basal *Cdk1* promoter activity but efficiently suppressed E2F1-dependent *Cdk1* promoter activity. Remarkably, Bmi1 almost completely relieved the necdin-induced suppression. Because Bmi1 directly binds to the *p16* promoter and downregulates its transcriptional activity [28], we examined the effect of necdin on *p16* promoter activity (Fig. 5D). Although necdin had little or no effect on basal *p16* promoter activity, necdin efficiently restored the Bmi1-induced suppression to the control level.

### Necdin Regulates NPC Proliferation and Expression of *p16* and *Cdk1* mRNAs

**Figure 6:**
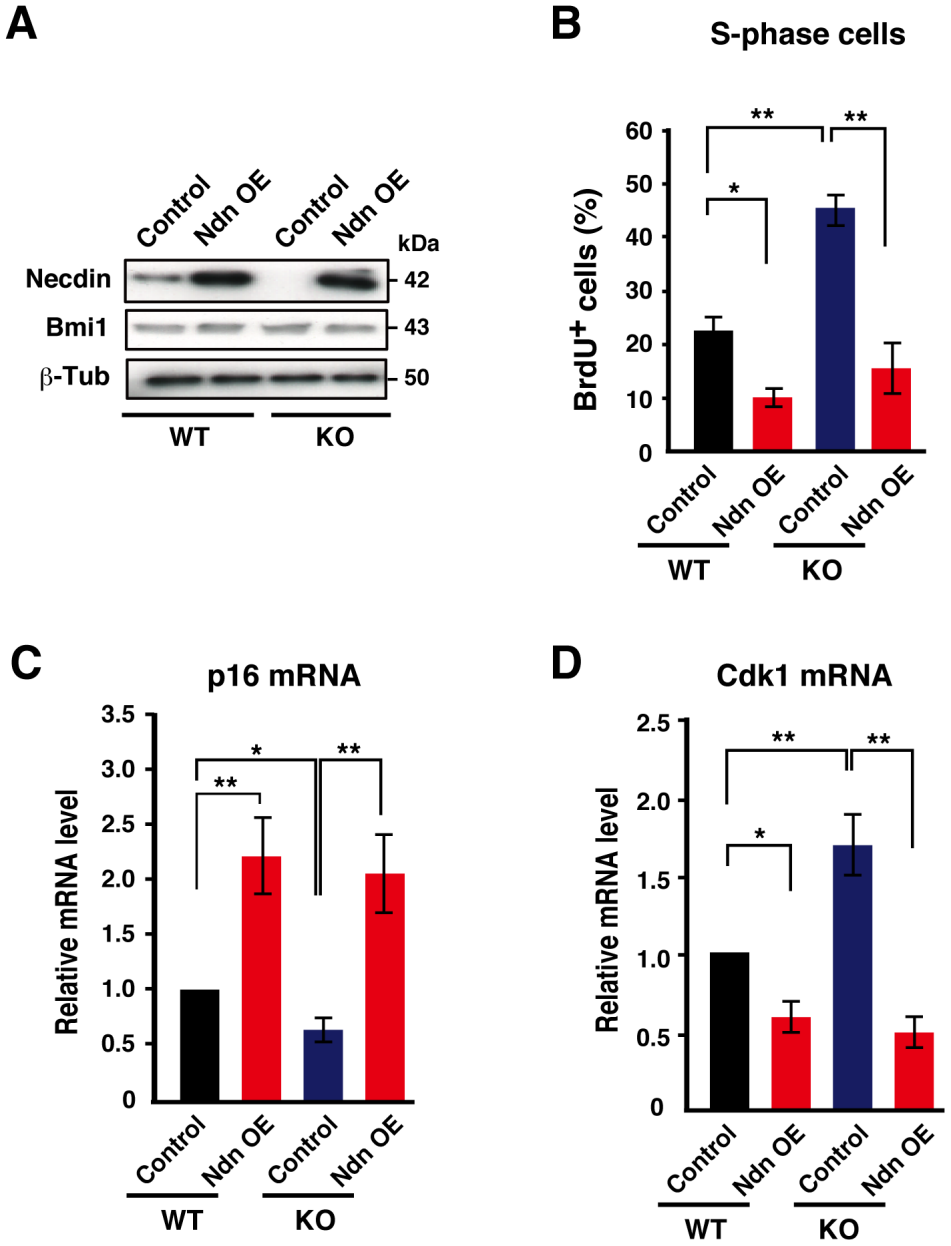
Necdin regulates NPC proliferation and expression of *p16* and *Cdk1* mRNAs. (**A**) Expression levels of necdin and Bmi1 in lentivirus-infected primary NPCs. Neocortical NPCs were prepared from wild-type (WT) and necdin-null (KO) mice at E14.5 and infected with lentiviruses for GFP expression (Control) and necdin overexpression (Ndn OE). Infected NPCs were cultured for 48 hrs and harvested for Western blot analysis of necdin, Bmi1, and β-tubulin (β-Tub). (**B**) Proliferation assay. The S-phase cell population was analyzed 48 hrs after viral infection by BrdU incorporation assay. (**C**, **D**) Expression levels of *p16* mRNA (**C**) and *Cdk1* mRNA (**D**) were determined by qRT-PCR. Values in (**B**−**D**) represent the mean ± SEM, *n = *3. *p<0.05, **p<0.01.

To examine whether the protein-protein interaction between necdin and Bmi1 regulates NPC proliferation, we used lentivirus-mediated gene transfer system to express necdin, Bmi1, and Bmi1 small hairpin RNA (shRNA) in primary NPCs. NPCs infected with a necdin-expressing lentivirus expressed high levels of the necdin protein as analyzed by Western blotting (Fig. 6A). We examined the effect of lentivirus-mediated necdin overexpression on proliferation of wild-type and necdin-null NPCs using BrdU incorporation assay (Fig. 6B). In wild-type NPCs, necdin overexpression reduced the BrdU^+^ cell population to 44% of the control level. The BrdU^+^ cell population of necdin-null NPCs increased 2-fold, and necdin overexpression reduced it by 66%. We next examined the effects of necdin overexpression on the expression levels of *p16* and *Cdk1* mRNAs in primary NPCs (Fig. 6C). In wild-type NPCs, necdin overexpression induced a 2.2-fold increase in the *p16* mRNA level. In necdin-null NPCs, the *p16* mRNA level decreased to 40% of the control level and was increased 3.5-fold by necdin overexpression. These results imply that necdin overexpression promotes *p16* transcription via Bmi1 repression in NPCs. In contrast, necdin overexpression reduced the *Cdk1* mRNA level by 40% in wild-type NPCs (Fig. 6D). In necdin-null NPCs, the *Cdk1* mRNA level was 1.7 times that of wild-type control level, and necdin overexpression reduced it by 71%. These results suggest that necdin efficiently downregulates *Cdk1* expression in neocortical NPCs.

### Bmi1 Regulates NPC Proliferation and Expression of *p16* and *Cdk1* mRNAs

**Figure 7:**
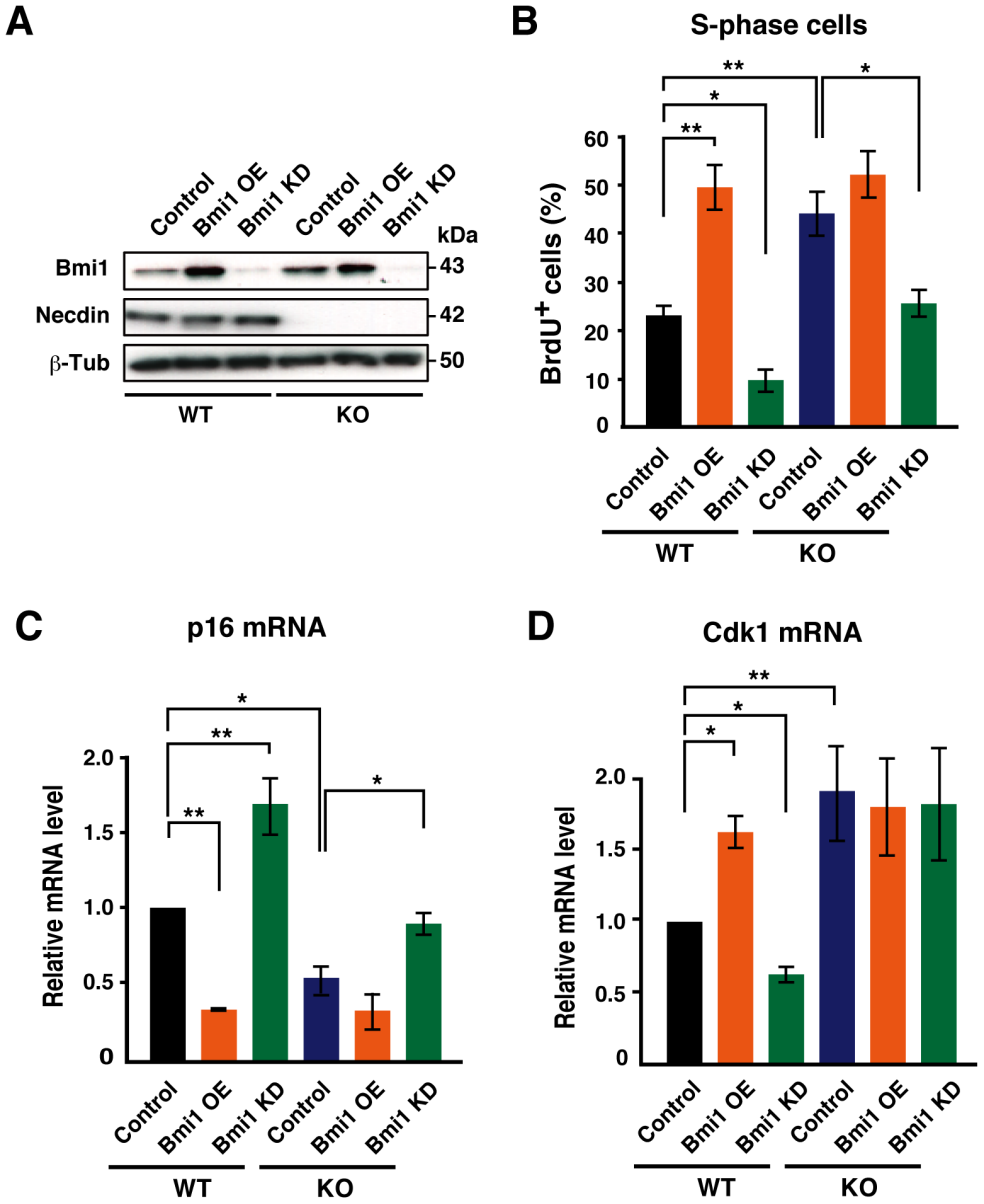
Bmi1 regulates NPC proliferation and expression of *p16* and *Cdk1* mRNAs. (**A**) Expression levels of necdin and Bmi1 in lentivirus-infected primary NPCs. Neocortical NPCs were prepared from wild-type (WT) and necdin-null (KO) mice at E14.5 and infected with lentiviruses for GFP expression (Control), Bmi1 overexpression (Bmi1 OE) and *Bmi1* shRNA expression (Bmi1 KD). Infected NPCs were cultured for 48 hrs and harvested for Western blot analysis of Bmi1, necdin, and β-tubulin (β-Tub). (**B**) Proliferation assay. The S-phase cell population was analyzed 48 hrs after viral infection by BrdU incorporation assay. (**C**, **D**) Expression levels of *p16* mRNA (**C**) and *Cdk1* mRNA (**D**) were determined by qRT-PCR. Values in (**B**−**D**) represent the mean ± SEM, *n = *3. *p<0.05, **p<0.01.

We determined the effects of Bmi1 overexpression and knockdown on the proliferation rates of neocortical NPCs. Western blot analysis revealed that the Bmi1 protein levels in primary NPCs infected with lentiviruses expressing Bmi1 and *Bmi1* shRNA were elevated and diminished, respectively (Fig. 7A). Neither Bmi1 overexpression nor Bmi1 knockdown affected endogenous necdin levels in wild-type NPCs. In wild-type NPCs, Bmi1 overexpression induced a 2.2-fold increase in the BrdU^+^ cell population, and Bmi1 knockdown decreased it by 58% (Fig. 7B). In necdin-null NPCs, Bmi1 overexpression had no significant effect on the BrdU^+^ cell population, whereas Bmi1 knockdown reduced it by 42%. We then examined the effects of Bmi1 overexpression and knockdown on the *p16* and *Cdk1* mRNA levels in NPCs. Bmi1 overexpression markedly reduced the *p16* mRNA level by 70%, whereas Bmi1 knockdown induced a 1.7-fold increase (Fig. 7C). The *p16* mRNA level was significantly reduced by 50% in necdin-null NPCs, in which Bmi1 knockdown increased it by 80%, indicating that endogenous Bmi1 downregulates p16 expression in a necdin-independent manner. In contrast, Bmi1 overexpression induced a 1.6-fold increase in the *Cdk1* mRNA level, whereas Bmi1 knockdown reduced it by 40% (Fig. 7D). In necdin-null NPCs, the Cdk1 level was 1.9-times the wild-type level, whereas neither Bmi1 overexpression nor Bmi1 knockdown exerted significant effects on the *Cdk1* mRNA level, suggesting that the regulatory effects of Bmi1 on *Cdk1* expression are mediated by endogenous necdin in NPCs.

Because Bmi1 downregulates the expression of *p19* mRNA [29], [30] and *p21* mRNA [31], [32] in NPCs, we quantified these mRNA levels in necdin-null NPCs (Fig. S6). There were no significant differences in the *p19* and *p21* mRNA levels between wild-type and necdin-null NPCs. Furthermore, necdin overexpression exerted no significant effects on these mRNA levels, suggesting that expression of *p19* and *p21* genes is regulated in a necdin-independent manner. In contrast, Bmi1 overexpression reduced the *p21* and *p19* mRNA levels by 40%, whereas Bmi1 knockdown induced 1.7- and 2.2-fold increases in the *p19* and *p21* mRNA levels, respectively, consistent with the previous reports [29]–[32] (Fig. S7). There were no significant differences in the degrees of these mRNA changes between wild-type and necdin-null NPCs.

## Discussion

**Figure 8:**
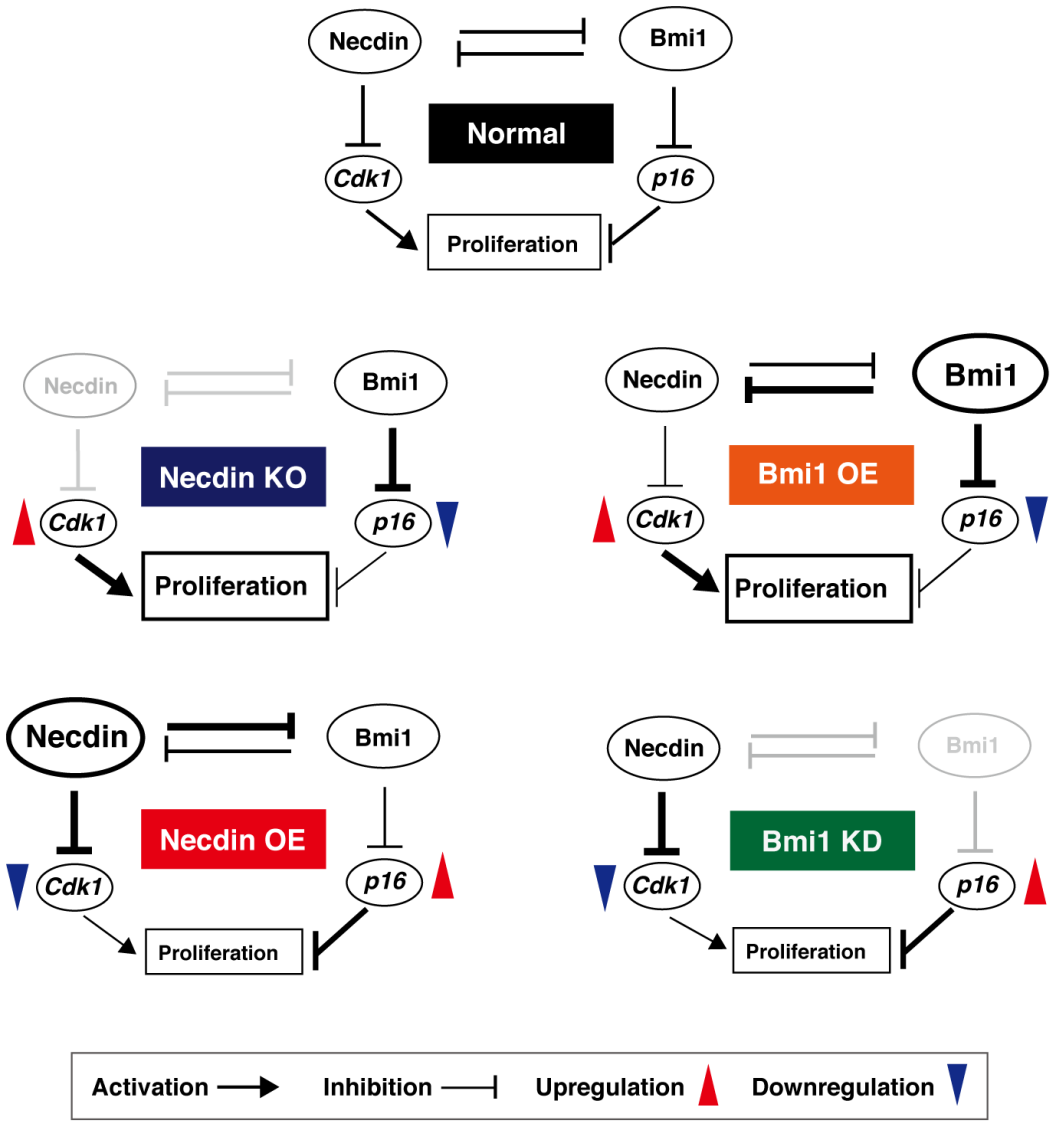
Antagonistic interplay between necdin and Bmi1. Data shown in Figures 6 and 7 were schematically presented. For details, see Discussion.

An accumulating body of evidence has suggested that Bmi1 is involved in NPC proliferation during embryonic and postnatal periods [23], [30]–[35]. The present study has shown that necdin and Bmi1 counteract each other to control proliferation of embryonic NPCs residing in the developing neocortex. On the basis of present data, we propose that the protein-protein interaction between necdin and Bmi1 controls NPC proliferation via Cdk1 and p16 pathways (Fig. 8). Under normal conditions, necdin, which suppresses *Cdk1* transcription, and Bmi1, which suppresses *p16* transcription, antagonize each other to regulate the proliferation of NPCs. In necdin-null NPCs (Necdin-KO), *Cdk1* expression is upregulated, whereas *p16* expression is downregulated via Bmi1 derepression, resulting in the enhancement of NPC proliferation. Bmi1 overexpression suppresses necdin and induces changes similar to those seen in necdin-null NPCs (Bmi1 OE). In necdin-overexpressing NPCs (Necdin OE), *Cdk1* expression is downregulated, whereas *p16* expression is upregulated via Bmi1 repression, resulting in the reduced NPC proliferation. Bmi1 knockdown (Bmi1 KD) suppresses *Cdk1* expression via necdin derepression and induces changes similar to those seen in necdin-overexpressing NPCs. We assume that these events in primary NPCs *in vitro* also occur in NPCs *in vivo* in the embryonic neocortex.

The present findings suggest that necdin and Bmi1 use their downstream cell-cycle regulatory systems via Cdk1 and p16 pathways to control NPC proliferation: Necdin suppresses Cdk1 expression (independently of Bmi1), increases p16 expression by repressing Bmi1, and suppresses NPC proliferation, whereas Bmi1 suppresses p16 expression (independently of necdin), increases Cdk1 expression by repressing necdin, and increases NPC proliferation. Necdin binds directly to E2F1 on the *Cdk1* promoter to suppress *Cdk1* transcription [13]. Cdk1 is essential for controlling cell divisions during embryonic development and executes all the cell cycle-related events even in the absence of interphase Cdks such as Cdk2, Cdk4, and Cdk6 [36]–[38]. Accordingly, necdin may exert its anti-mitotic effect by suppressing *Cdk1* expression at the transcription level in various cellular contexts. On the other hand, the proliferation capacity is reduced in Bmi1-deficient NPCs in which p16 expression is upregulated, and p16 deficiency reverses their proliferation defect [23], suggesting that endogenous Bmi1 upregulates NPC proliferation by repressing p16 expression. In the present study, p16 expression was significantly downregulated in necdin-null NPCs *in vivo* (Fig. 2). In necdin-null mice, the p16 immunoreactivity was markedly diminished in most neocortical cells but not in a specific cell population such as that undergoing apoptosis (Fig. 2D), suggesting that necdin upregulates p16 expression by repressing Bmi1 in a ubiquitous manner. Thus, necdin is likely to exert its anti-mitotic effect on embryonic NPCs by repressing Cdk1 expression and increasing p16 expression via Bmi1 repression.

Expression of the *necdin* gene (*Ndn*) is regulated by genomic imprinting, a mammal-specific epigenetic mechanism whereby certain genes are silenced in a parent-of-origin-specific manner [39], [40]. Human *NDN* is located in chromosome 15q11-12, a region responsible for the pathogenesis of the human neurodevelopmental disorder Prader-Willi syndrome. The maternal *NDN* allele is silenced through hypermethylation of CpG-rich sequences, and necdin is expressed only from the paternal *NDN* allele, whose deletion causes a complete defect of necdin expression. Bmi1, a component of the Polycomb repressive complex 1 that regulates gene silencing by remodeling the chromatin structure, affects gene silencing through epigenetic mechanism [41], [42]. However, the present study demonstrated that Bmi1 overexpression or knockdown exerted no significant effect on necdin expression in primary NPCs (Fig. 7A). This may be because acute lentivirus-mediated modulations of Bmi1 expression are insufficient to affect epigenetic controls of necdin expression. The previous study on the Bmi1-dependent transcriptional control of imprinted genes has shown that necdin expression is significantly derepressed in lung epithelial stem cells prepared from *Bmi1*-mutated mice [42]. These findings suggest that Bmi1 deficiency during critical periods for genomic imprinting is indispensable for the increase in necdin expression.

The present study has shown that necdin deficiency significantly increases the apoptotic cell population in embryonic neocortex *in vivo* and primary NPCs, indicating that necdin suppresses both proliferation and apoptosis of neocortical NPCs. It is noteworthy that *Drosophila* MAGE, a necdin-homologous MAGE protein, is expressed in neural stem cells (neuroblasts) and their progeny (ganglion mother cells and postmitotic neurons) in postembryonic neurogenesis [43], and that downregulation of MAGE expression in the developing mushroom bodies increases both NPC proliferation and neuronal apoptosis [44], implying that these evolutionally distant MAGE proteins possess the conserved function required for neurogenesis. The present data also demonstrate that necdin-null mice have an increased population of neocortical cells in the postnatal period (Fig. S4), suggesting that the increased proliferation rate of necdin-null NPCs exceeds the increased apoptosis rate. Thus, we suggest that necdin is indispensable for normal neocortical development in mammals by suppressing both proliferation and apoptosis of NPCs during the period of embryonic neurogenesis.

It has previously been demonstrated that necdin-null mice exhibit an enhanced number of proliferating hematopoietic stem cells during hematopoietic regeneration, suggesting that necdin prevents their excessive proliferation [18]. Similarly, necdin may exert its anti-mitotic effect on NPCs to prevent their excessive proliferation. It is also possible that necdin in NPCs is involved in terminal differentiation-associated withdrawal from the cell cycle. In this case, necdin deficiency may increase the number of differentiated postmitotic cells. We have previously found that necdin is moderately expressed in preadipocytes or mesenchymal stem cells residing in white adipose tissues, and that the number of terminally differentiated adipocytes is markedly increased in necdin-null mice fed a high-fat diet [20]. Similarly, the number of postmitotic neurons was slightly but significantly increased in the neocortex of necdin-null postnatal mice in the present study (Fig. S4). On the other hand, mitotically quiescent postmitotic neurons express higher levels of necdin than these proliferating stem cells or progenitors during mouse development [6]. We speculate that necdin prevents postmitotic neurons from aberrant re-entry into the cell cycle.

During development of necdin-null mice, apoptosis is enhanced in sensory neurons of the dorsal root ganglia [45], [46], granule neurons and their progenitors in the internal and external granule layers, respectively, of the cerebellum [13], and motor neurons of the spinal cord [47]. In the present study, the number of apoptotic cells was significantly increased only in the proliferative zone of necdin-null mice (Fig. S3). The apoptotic NPC population *in vivo* is much smaller in the neocortex than that in the GEs [21]. These findings indicate that the apoptosis rates in necdin-null mice vary depending on cell types and developmental stages. This may be because necdin interacts with various pro-apoptotic and pro-survival proteins that are expressed in a cell type and stage-specific manner [13], [21], [45]–[47]. Although the mechanism whereby necdin prevents apoptosis of neocortical NPCs remains to be elucidated, we speculate that necdin targets p53 which is highly expressed in neocortical NPCs *in vivo* [48]. Because necdin strongly suppresses p53-mediated neuronal apoptosis induced by DNA damage [49], we are currently investigating whether necdin suppresses p53-mediated apoptosis of neocortical NPCs.

Necdin, like Rb, interacts with the viral oncoproteins SV40 large T antigen and adenovirus E1A [9], suggesting that these viral proteins interact with endogenous necdin and inhibit its anti-mitotic activity to promote malignant transformation of infected cells. However, the mechanisms that regulate the function of necdin under physiological conditions have been poorly understood until recently. We have most recently reported that the necdin protein in GE-derived NPCs is degraded under hypoxic conditions by hypoxia-inducible factor-2α via the ubiquitin-proteasome pathway [21], implying that the function of necdin in NPCs is modulated in an oxygen tension-dependent manner. The present study has demonstrated another mechanism whereby Bmi1 suppresses necdin by the direct protein-protein interaction. These findings suggest that multiple pathways that control the activity of endogenous necdin are operative in embryonic NPCs to maintain their proper proliferation rates. Thus, we propose that necdin serves as a molecular rheostat to regulate the expansion of embryonic NPCs.

## Materials and Methods

### Ethics Statement

This study was approved by the Animal Experiment Committee (Approval No. 24-04-0) and Recombinant DNA Committee (Approval No. 2938-1) of Institute for Protein Research, Osaka University, and performed in accordance with national and institutional guidelines for the care and use of animals. All efforts were made to minimize the number of animals and their suffering. Mice were sacrificed by cervical dislocation or decapitation according to the American Veterinary Medical Association Guidelines for the Euthanasia of Animals 2013.

### Animals

*Ndn* mutant mice (*Ndn* ^tm1Ky^) were generated and maintained as described in [45]. Heterozygous male mice (*Ndn* ^+/−^) (>20 generations in ICR background) were crossed with wild-type female mice (*Ndn* ^+/+^) to obtain wild-type (*Ndn* ^+m/+p^) and paternal *Ndn*-deficient (*Ndn* ^+m/−p^) littermates. All mice were housed in a 12-hour light/dark cycle with room temperature at 23±3°C. Pregnant female mice at gestation day 14.5 were sacrificed by cervical dislocation, and embryos were collected. Genotypes of all mice were analyzed by PCR for mutated Ndn locus. Experiments using gene-targeted mice were approved by the Recombinant DNA and Animal Experiment Committees of the Institute for Protein Research, Osaka University, and performed in accordance with institutional guidelines and regulations.

### Immunohistochemistry

Brain tissues of mouse embryos at embryonic day 14.5 (E14.5) were fixed with 4% paraformaldehyde in phosphate buffer (pH 7.4) overnight, and cryoprotected by immersion in 20% sucrose overnight. Frozen 10 µm-thick tissue sections were immunostained as described in [7]. For 5′-bromo-2′-deoxyuridine (BrdU) labeling, pregnant dams were injected intraperitoneally with BrdU (50 mg/kg body weight, Sigma-Aldrich) at gestation day 14.5, and the embryonic forebrain was prepared 4 hrs later. For detection of Sox2, phospho-histone H3 (pH3), and p16, cryosections were subjected to antigen retrieval using a microwave oven (MI-77, Azumaya) for 20 min at 90°C in 0.01 M sodium citrate pH 6.0. Sections were incubated with primary antibodies at 4°C overnight and fluorescence dye-conjugated secondary antibodies at room temperature for 90 min. The primary antibodies used are: necdin (GN1; 1∶500) [45], Sox2 (R&D Systems; 1∶300), Nestin (ST-1; 1∶1000) [50], βIII-tubulin (Promega; 1∶1000), pH3 (Merck Millipore; 1∶300), BrdU (Abcam; 1∶300), p16 (Santa Cruz F-12; 1∶300). The secondary antibodies were cyanine 3-conjugated anti-guinea pig IgG (Jackson ImmunoResearch; 1∶500), Alexa 488-conjugated anti-mouse IgG (Life Technologies; 1∶500), Alexa 488-conjugated anti-rabbit IgG (Life Technologies; 1∶500), cyanine 3-conjugated anti-rat IgG (Jackson ImmunoResearch; 1∶500). The images were observed with a fluorescence microscope (BX-34-FLAD1; Olympus), taken by charge-coupled device (CCD) camera system (DP-70; Olympus), and processed by Adobe Photoshop CS5 software. For fluorescence microphotometry, fluorescence images (12-bit digital monochrome images) were captured with a CCD camera (CoolSNAP monochrome, Nippon Roper) and analyzed using a fluorescence image analysis software (Fluoroimage Cool V, Mitani) [49]. The background intensity of the equal-sized adjacent area without signals was subtracted from the intensity of signal-containing area. For quantification of positive cells or fluorescence signals, 5 consecutive 10-µm-thick sections per embryo were analyzed.

### Quantitative RT-PCR

Total RNA was extracted with phenol and guanidine thiocyanate mixture (TRI Reagent, Molecular Research Center), and contaminating DNA was digested with RQ1 RNase-free DNAase (Promega). cDNA was synthesized from total RNA (2 µg) using Transcriptor First Strand cDNA Synthesis Kit (Roche). An aliquot of cDNA mixture (0.2%) was used as PCR templates. Primers used for quantitative real-time PCR are as follows: p16 (forward, 5′-gccgtgtgcatgacgtgcgg-3′; reverse, 5′-gtcctcgcagtttcgaatctgcac-3′), p19 (forward, 5′-aggttcttggtcactgtgagg-3′; reverse, 5′-gaatctgcaccgtagttgagc-3′), p21 (forward, 5′-ggacaagaggcccagtacttc-3′; reverse, 5′-atctgcgcttggagtgataga-3′), p27 (forward, 5′-acgccagacgtaaacagctccgaa-3′; reverse, 5′-acaacctaattgcgcaatgctacatcca-3′), p57 (forward, 5′-actgctgcggccaatgcgaa-3′; reverse, 5′-ccgaagcccagagttcttccatcgt-3′), Cdk1 (forward, 5′-atggaagaggaccaactgtc-3′; reverse, 5′-gagccaacggtaaacaacac-3′), Sox2 (forward, 5′-caatcccatccaaattaacgca-3′; reverse, 5′-aagctgcagaatcaaaaccc-3′), Necdin (forward, 5′-aggacctgagcgaccctaac-3′; reverse, 5′-tgctgcaggattttagggtcaac-3′), Bmi1 (forward, 5′-cagcaatgactgtgatgc-3′; reverse, 5′-ctccagcattcgtcagtc-3′), Gapdh (forward, 5′-gaatacggctacagcaacag-3′; reverse, 5′-gcagcgaactttattgatggta-3′). RT-PCR products were quantified using FastStart DNA MasterPLUS SYBR Green I kit (Roche Diagnostics) and a real-time PCR instrument (LightCycler Roche Diagnostics). Melting curves were analyzed to confirm a single species of each PCR product. Gapdh cDNA was used as an internal control to quantify the relative expression of each cDNA.

### Western Blotting

Neocortical tissues of E14.5 mice and cultured cells were homogenized with lysis buffer containing 10 mM Tris-HCl, pH 8.0, 150 mM NaCl, 1 mM EDTA, 1% Nonidet P-40, 10% glycerol, and a protease inhibitor mixture (Complete; Roche). For subcellular distribution analysis, cytoplasmic and nuclear fractions of cultured NPCs were separated as described in [51]. The protein concentration was determined by the Bradford method (Bio-Rad). The lysates (10 µg protein per lane) were separated by 10% SDS-PAGE, electroblotted to polyvinylidene difluoride membranes (Immobilon, Merck Millipore), and incubated with primary antibodies. The primary antibodies used are: p16 (Santa Cruz F-12; 1∶200), Cdk1 (Santa Cruz sc-54; 1∶300), Sox2 (R&D Systems; 1∶300), necdin (NC243; 1∶1000) [52], β-tubulin (Sigma-Aldrich; 1∶1000), Bmi1 (GBmi1; 1∶300; raised in guinea pig against GST-fused full length Bmi1), Lamin B (Santa Cruz C-20; 1∶500), Nestin (ST-1; 1∶1000), Myc (9E10; 1∶10), proliferating cell nuclear antigen (PCNA) (Santa Cruz PC10, 1∶1000), and necdin (GN1; 1∶1000). After incubation with peroxidase-conjugated IgGs (Cappel), the proteins were detected by chemiluminescence method (Chemiluminescence Reagent Plus; PerkinElmer). Signal intensities were quantified by ImageJ 1.44 software. β-tubulin was used as an internal control to normalize the expression level of each protein.

### Primary NPCs

Neocortical tissues dissected from E14.5 mice were treated with 0.05% trypsin, and dissociated cells were incubated at 37°C for 6 hrs in a basal medium containing DMEM/F12 medium (Life Technologies), 14 mM sodium bicarbonate, 1 mM N-acetyl-L-cysteine, 33 mM D (+)-glucose and 1 mg/ml bovine serum albumin. Floating NPCs were separated from non-NPCs attached on the culture dish bottom and collected. Primary NPCs were plated onto 35-mm dishes (2.5–5×10^5^ cells per dish) and incubated in the basal medium supplemented with 2 mM L-glutamine, 20 ng/ml EGF (PeproTech), 20 ng/ml bFGF (PeproTech) and B-27 supplement (1∶50 dilution) (Life Technologies) for 48 hrs at 37°C under humidified 5% CO_2_ conditions. For NPC differentiation, NPCs were cultured in the basal medium supplemented with 2 mM L-glutamine, B-27 supplement (1∶50 dilution) and 3% fetal bovine serum (FBS). Primary neurons were prepared from the neocortex at E14.5 and incubated in Neurobasal medium (Life Technologies) supplemented with 2 mM L-glutamine and B-27 supplement (1∶50 dilution) as described in [49].

### Immunocytochemistry

Primary NPCs were dispersed by trypsin treatment, plated onto 35-mm dishes or 12-well plates precoated with poly-L-ornithine, and cultured for 24 hrs. Cells were fixed with 10% formalin solution at room temperature for 20 min and permeabilized with methanol at room temperature for 20 min. Fixed cells were incubated with primary antibodies at 4°C overnight, and with secondary antibodies at room temperature for 90 min. The primary antibodies used are: necdin (GN1; 1∶500), Sox2 (R&D Systems; 1∶300), nestin (ST-1; 1∶1000), βIII-tubulin (Promega; 1∶1000), GFAP (1∶1000) [50], BrdU (Abcam; 1∶500), necdin (NC243; 1∶500) [52], Bmi-1 (Merck Millipore; 1∶300), and Green Fluorescent Protein (GFP) (1∶500; MBL). The secondary antibodies are: cyanine 3-conjugated guinea pig IgG (Life Technologies; 1∶500), Alexa 488-conjugated anti-mouse IgG (Life Technologies; 1∶500), Alexa 488-conjugated anti-rabbit IgG (Life Technologies; 1∶500), cyanine 3-conjugated anti-rabbit IgG (Jackson ImmunoResearch; 1∶500), cyanine 3-conjugated anti-rat IgG (Jackson ImmunoResearch; 1∶500), and Alexa 488-conjugated anti-guinea pig IgG (Life Technologies; 1∶500). Chromosomal DNA was stained with 5 µM Hoechst 33342 (Sigma-Aldrich). Immunofluorescence images were observed by fluorescence microscopy and processed using Adobe Photoshop CS5 software.

### Cell Proliferation Assay

NPCs were plated on coverslips in 12-well plates (1×10^5^ cells per well), cultured for 24 hrs, incubated for another 4 hrs in the presence of BrdU (10 µM), and fixed with 70% ethanol containing 20 mM glycine-HCl (pH 2.0) for 30 min at −20°C. For lentivirus-infected NPCs, BrdU was added 48 hrs after viral infection, and NPCs were incubated for another 4 hrs and fixed for immunocytochemistry. BrdU was detected by fluorescence immunocytochemistry as described in [20], [26]. For transfected cells, HEK293A cells were plated on coverslips in 35-mm dishes, cultured in DMEM supplemented with 10% FBS, and transfected with pEGFP-C2 (1 µg) (Clontech), pcDNA3.1+ carrying necdin cDNA (0.5 µg) and 6×Myc-Bmi1 cDNA (5 µg). The empty vector pcDNA3.1+ was added to equalize the amounts of transfected DNA (6 µg/assay). BrdU (10 µM) was added to the medium 48 hrs after transfection, and the cells were fixed 4 hrs later. BrdU and GFP were detected by fluorescence immunocytochemistry. For cell proliferation assay *in vivo*, pregnant mice were injected i.p. with 5′-ethynyl-2′-deoxyuridine (EdU, Life Technologies) (50 mg/kg body weight) at gestation day 14.5. Mice were sacrificed at P4 by decapitation, and forebrain tissues were fixed for EdU histochemistry. Incorporated EdU was detected using Click-iT EdU Alexa Fluor Imaging Kit (Life Technologies).

### Apoptosis Assay

Nuclear DNA fragmentation was analyzed by terminal deoxynucleotidyl transferase-mediated dUTP nick end labeling (TUNEL) visualized with Texas Red as described in [53], [54]. Primary NPCs were incubated for 48 hrs, dispersed, and fixed 24 hrs later. TUNEL analysis in brain tissues was performed as described in [54]. TUNEL^+^ cells in each 200-µm-wide column of the neocortex were counted.

### Co-immunoprecipitation Assay

Full-length Bmi1 cDNA (NCBI NM 007552.4) was synthesized from mouse E14.5 forebrain mRNA by RT-PCR. cDNAs encoding Bmi1 and its deletion mutants were subcloned into 6×Myc-pcDNA3.1+ [49]. Combinations of the expression vectors for necdin, 6×Myc-Bmi1, 6×Myc-Bmi1-deletion mutants, p53 and p53ΔN were transfected into HEK293A cells (Life Technologies) with calcium phosphate and harvested 24 hrs after transfection. Cell extracts (200 µg) were incubated at 4°C for 2 hrs with anti-necdin (NC243; 1∶100) and anti-Myc (9E10; 1∶4) antibodies in a lysis buffer containing 10 mM Tris-HCl, pH 8.0, 150 mM NaCl, 1 mM EDTA, 1% NP-40, 10% glycerol and 1×protease inhibitors. The complexes were pelleted with protein A-Sepharose (GE Healthcare), separated by 10% SDS-PAGE, and detected by Western blotting. For detection of endogenous necdin-Bmi1 complex, lysates of primary NPCs (500 µg protein) from E14.5 mice were incubated with rabbit anti-necdin IgG or preimmune IgG. Bound proteins were pelleted with Dynabeads protein A (Life Technologies) and detected by Western blotting as described in [49].

### *In vitro* Binding Assay

cDNAs encoding Bmi1 and its deletion mutants were subcloned into pCold-GST (Takara), and glutathione-S-transferase fusion proteins were synthesized in E. coli BL21 as described in [55]. The GST-Bmi1 fusions immobilized on glutathione Sepharose 4B (100 µl) (GE Healthcare) were incubated with His-tagged necdin (200 ng) at 4°C for 2 hrs in 1 ml of 50 mM Tris buffer (pH 8.0) containing 300 mM KCl and 2 mM 2-mercaptoethanol as described in [26]. After washing with 50 mM HEPES buffer (pH 8.0) containing 300 mM KCl and 1 mM DTT, bound proteins were eluted and detected by Coomassie Brilliant Blue staining. Bound His-tagged necdin was detected by Western blotting.

### Promoter Assay

The mouse *p16* promoter (−950 to +18, transcription start site = +1) was obtained by PCR using mouse genomic DNA and primers (forward, 5′-gatatctctgtctgcagcggactcca-3′; reverse, 5′-gagctcctggtaactctgcccaaagcgt-3′) and cloned into the pGL4 reporter vector (Promega). The *Cdk1* promoter (−498 to +46) was inserted into the luciferase reporter vector PGV-B (Toyo Ink) [13]. cDNAs encoding 6×Myc-Bmi1, E2F1, necdin were co-transfected with calcium phosphate into HEK293A cells. Luciferase activities were measured by a luminometer (Lumat LB9501, Berthold) using Dual-Luciferase Reporter Assay system (Promega). Transfection efficiency was normalized with the activity of coexpressed Renilla luciferase.

### Lentivirus Vectors

Recombinant lentiviruses were produced in HEK293FT cells transfected with SIN vector plasmids and two helper plasmids as described in [20], [56]. Necdin and Bmi1 cDNAs were subcloned into pENTR1A entry vector (Life Technologies) to construct the destination vectors CSII-EF1α-necdin-IRES-EmGFP and CSII-EF1α-Bmi1-IRES-EmGFP, in which Emerald Green Fluorescent Protein (EmGFP) (Life Technologies) was contained for an expression indicator. The lentivirus vector expressing Bmi1 shRNA CSII-EF1α-EmGFP-Bmi1 shRNA was constructed using BLOCK-iT Pol II miR RNAi Expression Vector Kits (Life Technologies) and double-stranded oligonucleotides described in [31]. The viral titer was measured by serial dilution on HEK293FT cells (Life Technologies) and determined as GFP^+^ cell population by immunocytochemistry. For viral transduction, lentiviral vectors were added to primary NPC cultures at a multiplicity of infection (moi) of 10, and the infected cells were incubated for 48 hrs prior to analyses.

### Statistics

Statistical significance was tested using an unpaired Student’s *t* test or one-way ANOVA followed by Tukey’s post hoc test. A significance of *p*<0.05 was required for rejection of the null hypothesis.

## Supporting Information

Figure S1 **Necdin deficiency increases pH3^+^ cells in the neocortex at E12.5 and E16.5.** (**A**, **B**) pH3^+^ cells at E12.5. Forebrain cryosections prepared from wild-type (WT) and necdin-null (KO) E12.5 mice were immunostained for pH3 (**A**). Chromosomal DNA was stained with Hoechst 33342 (blue), and pH3^+^ cells (green) within a 200-µm-wide radial column were counted (**B**). (**C**, **D**) pH3^+^ cells at E16.5. Cryosections prepared from E16.5 mice were analyzed as in (**A**, **B**). Abbreviations: PP, preplate; CP, cortical plate; IZ, intermediate zone; SVZ, subventricular zone; VZ, ventricular zone. Values represent the mean ± SEM, *n = *3; *p<0.05, **p<0.01. Scale bars, 50 µm.
(TIF)

Figure S2 **Neocortical thickness of wild-type and necdin-null mice.** Forebrain cryosections prepared from wild-type (WT) and necdin-null (KO) mice at E14.5 were stained with Hoechst 33342 (**A**), and the neocortical thickness between the apical surface of the VZ and the pial surface was measured (**B**). Values represent the mean ± SEM, *n = *3 (**C**). NS, not significant (*p*>0.05). Cortical areas are labeled as in Fig. S1. Scale bars; 250 µm in (**A**), 25 µm in (**B**).
(TIF)

Figure S3 **Necdin deficiency increases apoptosis of neocortical cells at different embryonic stages.** Frozen forebrain sections of E12.5 (**A**, **B**), E14.5 (**C**,**D**), E16.5 (**E**, **F**) mice were analyzed by TUNEL (**A**, **C**, **E**), and TUNEL^+^ cells within 200-µm-wide VZ (or VZ/SVZ), IZ and CP areas (labeled as in Fig. S1) were counted (**B**, **D**, **F**). Coronal cryosections of WT and KO mouse forebrain tissues at each embryonic stage were selected on the basis of their morphological similarities and were processed simultaneously for immunohistochemistry. Values represent the mean ± SEM, *n = *3. *p<0.05, **p<0.01. Scale bars, 50 µm.
(TIF)

Figure S4 **Distribution of E14.5-born neurons in the postnatal neocortex.** Pregnant female mice were injected with EdU at gestational day 14.5, and the forebrains of wild-type (WT) and necdin-null (KO) mice were prepared at P4. EdU^+^ neurons were detected by Alexa Fluor fluorescence (green) after staining DNA with Hoechst 33342 (blue) (**A**). HE, hematoxylin-eosin stain. Neocortical layers I-VI were divided into 10 equal bins, and the number of EdU^+^ cells in each bin was counted within each 500-µm-wide column (boxed in upper panels) after threshold setting (**B**). Scale bars; 500 µm (upper panel), 100 µm (lower panels) in (**A**). Arrows and arrowheads point to representative cells with high and low fluorescence intensities, respectively. Values represent the mean ± SEM, *n = *3. *p<0.05, **p<0.01.
(TIF)

Figure S5 **Necdin and Bmi1 mRNA levels in primary NPCs and neurons.** Primary NPCs and neurons were prepared from the neocortex of E14.5 wild-type (WT) and necdin-null (KO) mice. The necdin (**A**) and Bmi1 (**B**) mRNA levels were analyzed by qRT-PCR. Values represent the mean ± SEM, *n = *3.
(TIF)

Figure S6 **Necdin exerts no modulatory effects on** ***p19*** **and** ***p21*** **expression in neocortical NPCs.** Neocortical NPCs prepared from wild-type (WT) and necdin-null (KO) mice at E14.5 were infected with lentiviruses for GFP expression (Control) and necdin overexpression (Ndn OE). The *p19* (**A**) and *p21* (**B**) mRNA levels in lentivirus-infected NPCs were analyzed by qRT-PCR. Values represent the mean ± SEM, *n = *3. NS, not significant (*p*>0.05).
(TIF)

Figure S7 **Bmi1 exerts modulatory effects on** ***p19*** **and** ***p21*** **expression in neocortical NPCs.** Neocortical NPCs prepared from wild-type (WT) and necdin-null (KO) mice at E14.5 were infected with lentiviruses for GFP expression (Control), Bmi1 overexpression (Bmi1 OE), and *Bmi1* shRNA (Bmi1 KD). The *p19* (**A**) and *p21* (**B**) mRNA levels in lentivirus-infected NPCs were analyzed by qRT-PCR. Values represent the mean ± SEM, *n* = 3. *p<0.05, **p<0.01.
(TIF)
