# Supplementary figures and images for: Antagonistic Interplay between Necdin and Bmi1 Controls Proliferation of Neural Precursor Cells in the Embryonic Mouse Neocortex
Source: PLoS One. 2014 Jan 2;9(1):e84460. doi: 10.1371/journal.pone.0084460 (PMC3879318; doi:10.1371/journal.pone.0084460)

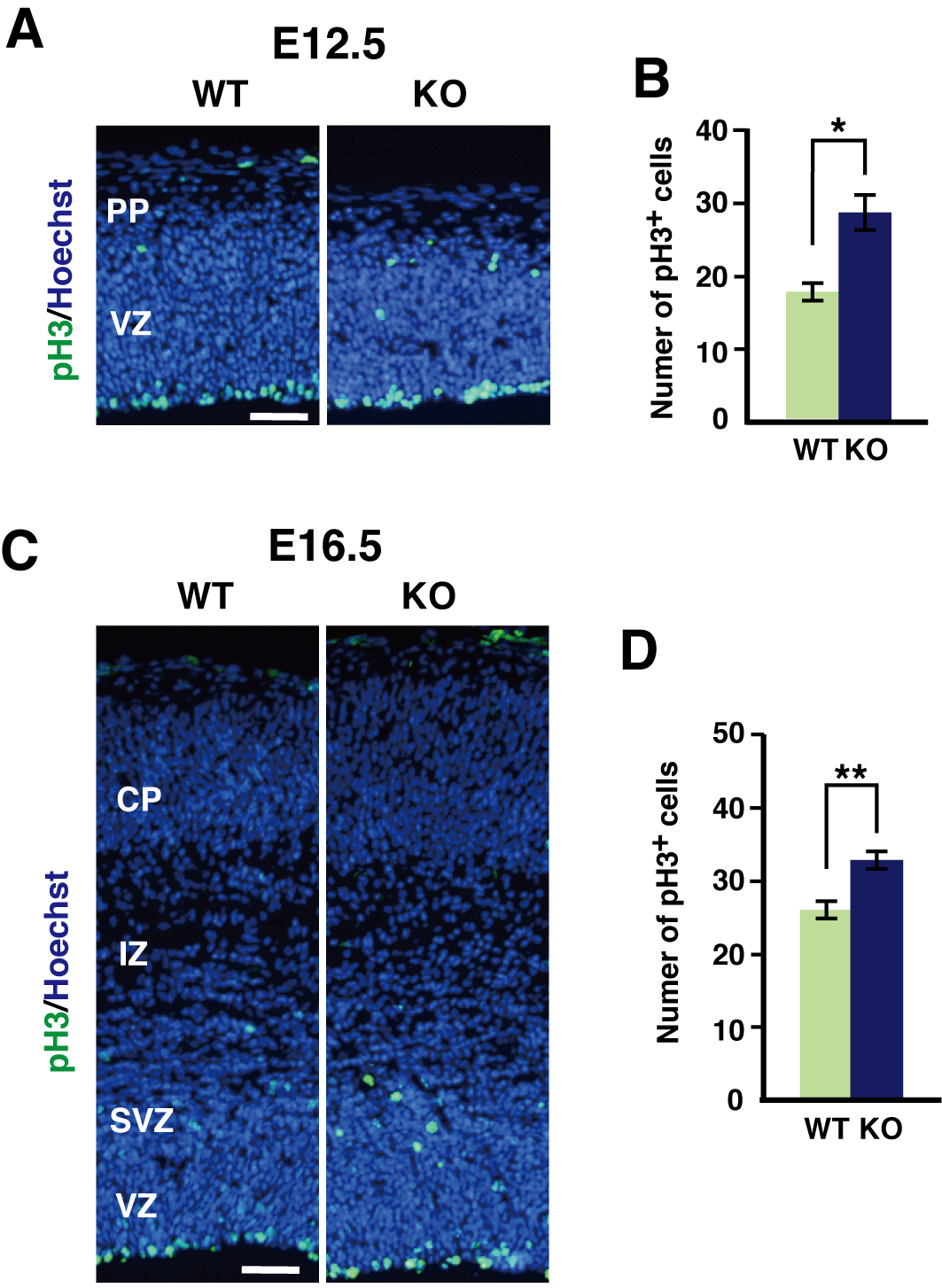

Supplement: Figure S1 — Necdin deficiency increases pH3+ cells in the neocortex at E12.5 and E16.5. (A, B) pH3+ cells at E12.5. Forebrain cryosections prepared from wild-type (WT) and necdin-null (KO) E12.5 mice were immunostained for pH3 (A). Chromosomal DNA was stained with Hoechst 33342 (blue), and pH3+ cells (green) within a 200-µm-wide radial column were counted (B). (C, D) pH3+ cells at E16.5. Cryosections prepared from E16.5 mice were analyzed as in (A, B). Abbreviations: PP, preplate; CP, cortical plate; IZ, intermediate zone; SVZ, subventricular zone; VZ, ventricular zone. Values represent the mean ± SEM, n = 3; *p<0.05, **p<0.01. Scale bars, 50 µm. (TIF) [file pone.0084460.s001.tif]

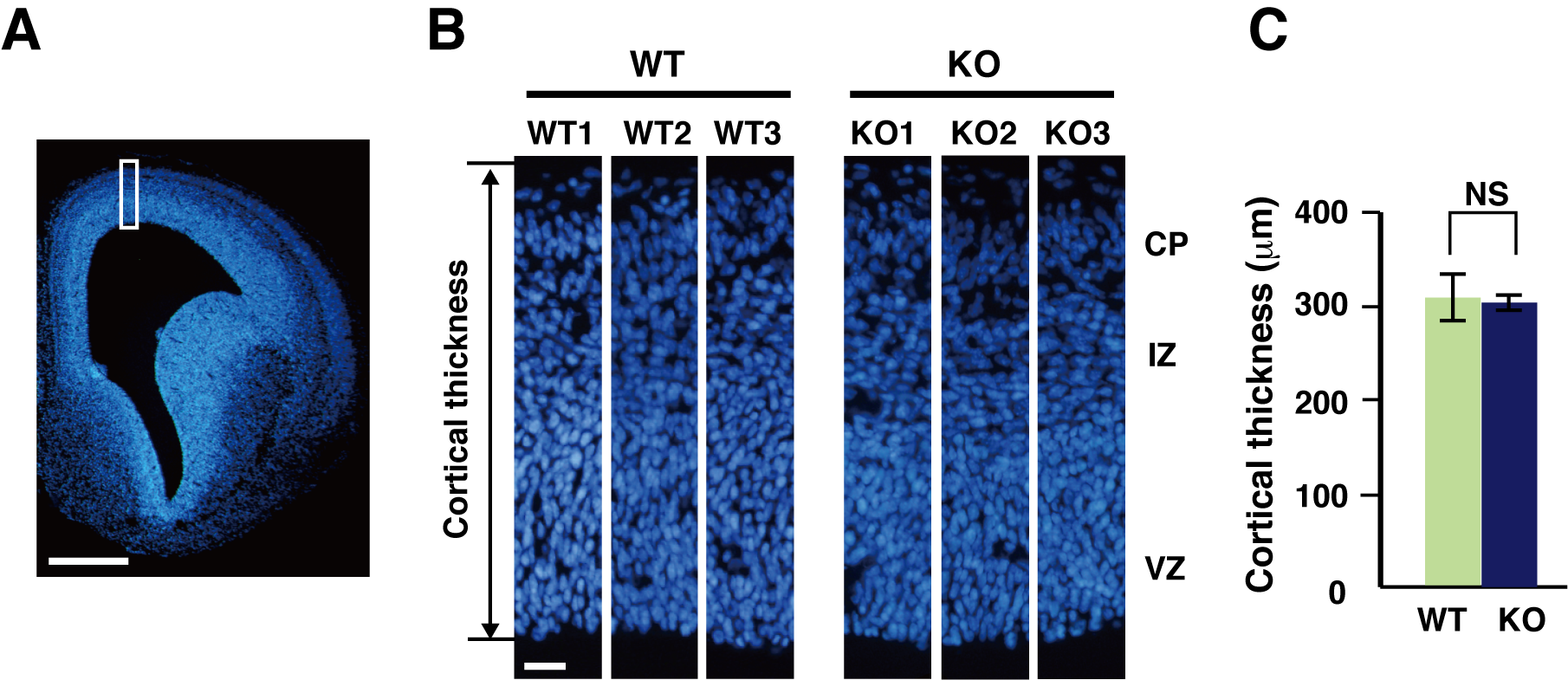

Supplement: Figure S2 — Neocortical thickness of wild-type and necdin-null mice. Forebrain cryosections prepared from wild-type (WT) and necdin-null (KO) mice at E14.5 were stained with Hoechst 33342 (A), and the neocortical thickness between the apical surface of the VZ and the pial surface was measured (B). Values represent the mean ± SEM, n = 3 (C). NS, not significant (p>0.05). Cortical areas are labeled as in Fig. S1. Scale bars; 250 µm in (A), 25 µm in (B). (TIF) [file pone.0084460.s002.tif]

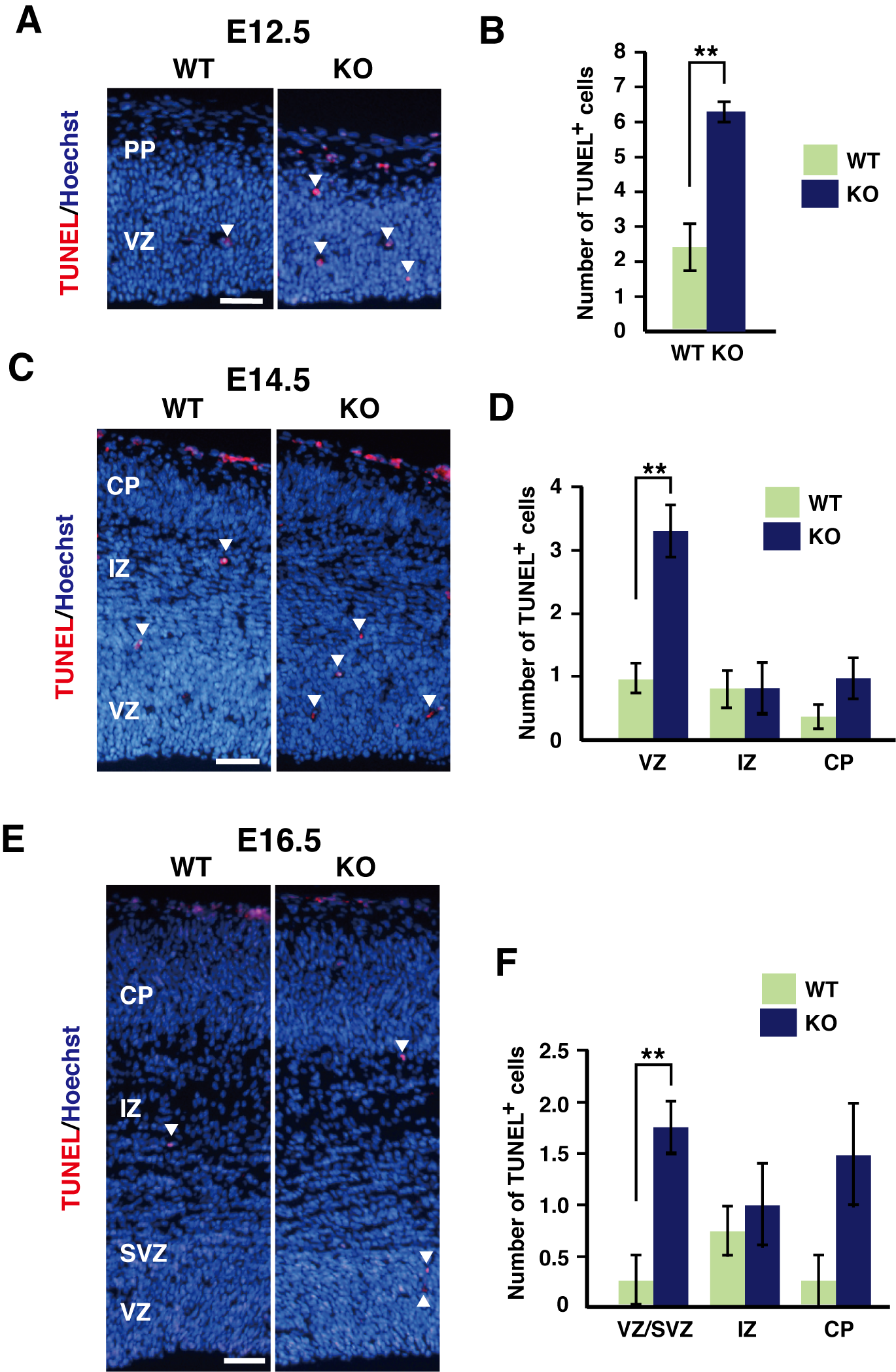

Supplement: Figure S3 — Necdin deficiency increases apoptosis of neocortical cells at different embryonic stages. Frozen forebrain sections of E12.5 (A, B), E14.5 (C,D), E16.5 (E, F) mice were analyzed by TUNEL (A, C, E), and TUNEL+ cells within 200-µm-wide VZ (or VZ/SVZ), IZ and CP areas (labeled as in Fig. S1) were counted (B, D, F). Coronal cryosections of WT and KO mouse forebrain tissues at each embryonic stage were selected on the basis of their morphological similarities and were processed simultaneously for immunohistochemistry. Values represent the mean ± SEM, n = 3. *p<0.05, **p<0.01. Scale bars, 50 µm. (TIF) [file pone.0084460.s003.tif]

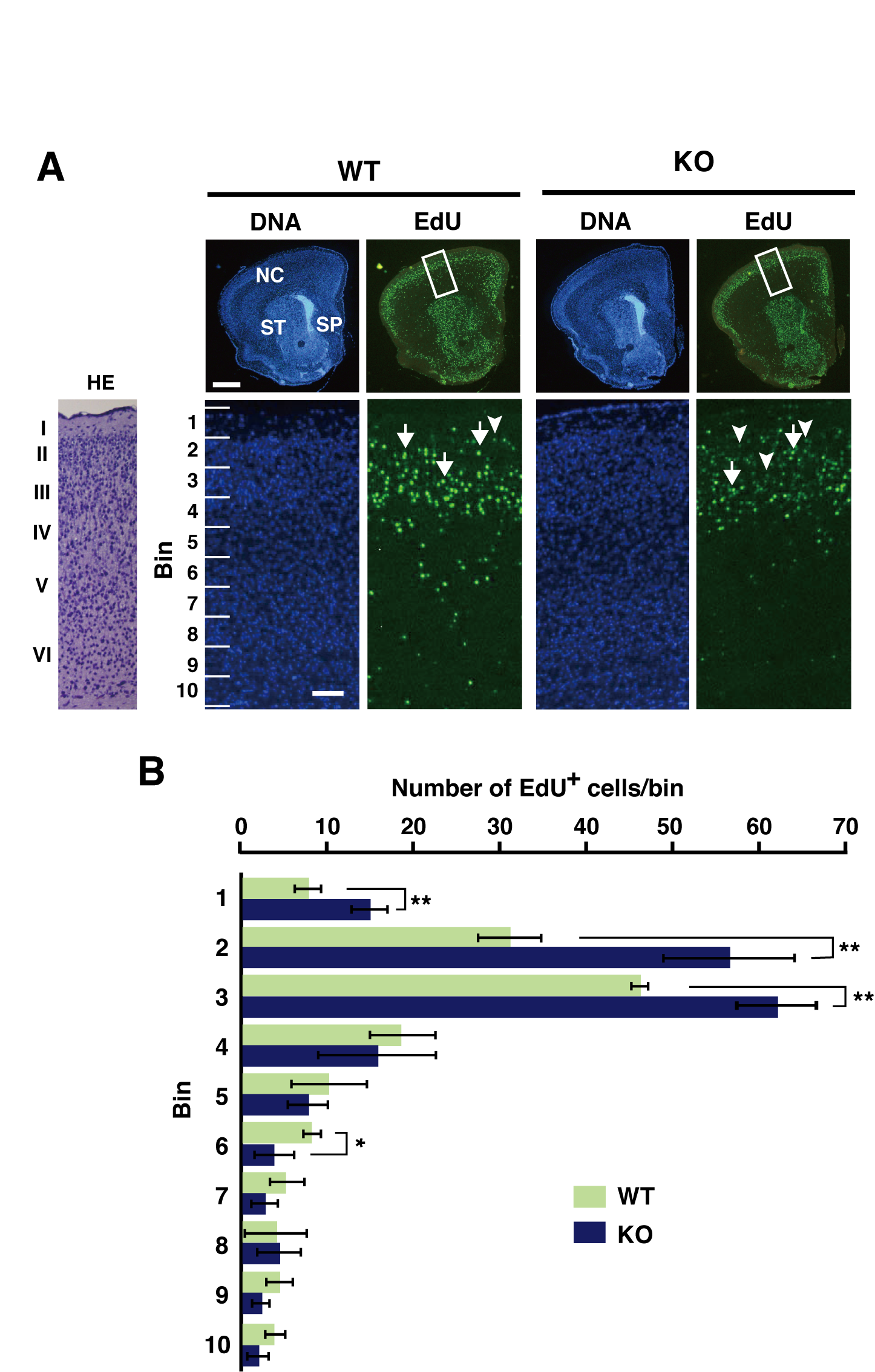

Supplement: Figure S4 — Distribution of E14.5-born neurons in the postnatal neocortex. Pregnant female mice were injected with EdU at gestational day 14.5, and the forebrains of wild-type (WT) and necdin-null (KO) mice were prepared at P4. EdU+ neurons were detected by Alexa Fluor fluorescence (green) after staining DNA with Hoechst 33342 (blue) (A). HE, hematoxylin-eosin stain. Neocortical layers I-VI were divided into 10 equal bins, and the number of EdU+ cells in each bin was counted within each 500-µm-wide column (boxed in upper panels) after threshold setting (B). Scale bars; 500 µm (upper panel), 100 µm (lower panels) in (A). Arrows and arrowheads point to representative cells with high and low fluorescence intensities, respectively. Values represent the mean ± SEM, n = 3. *p<0.05, **p<0.01. (TIF) [file pone.0084460.s004.tif]

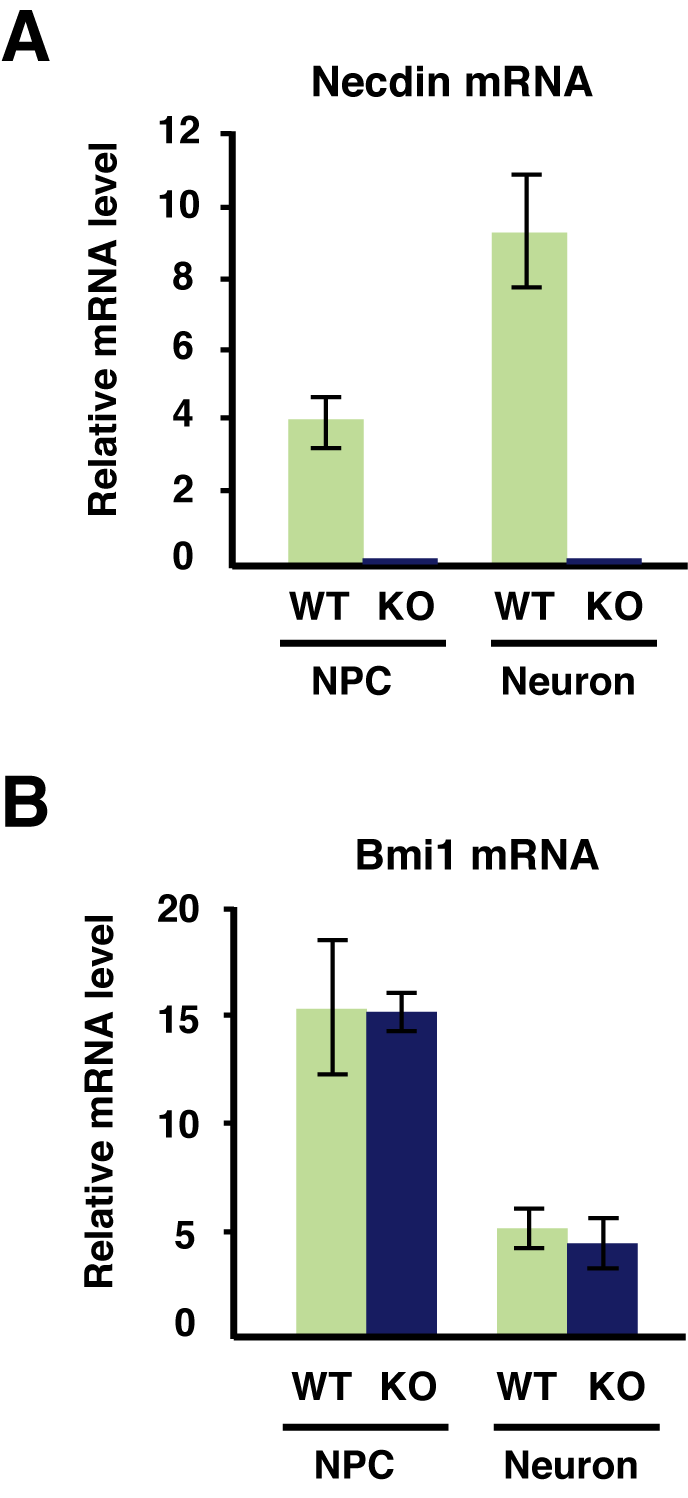

Supplement: Figure S5 — Necdin and Bmi1 mRNA levels in primary NPCs and neurons. Primary NPCs and neurons were prepared from the neocortex of E14.5 wild-type (WT) and necdin-null (KO) mice. The necdin (A) and Bmi1 (B) mRNA levels were analyzed by qRT-PCR. Values represent the mean ± SEM, n = 3. (TIF) [file pone.0084460.s005.tif]

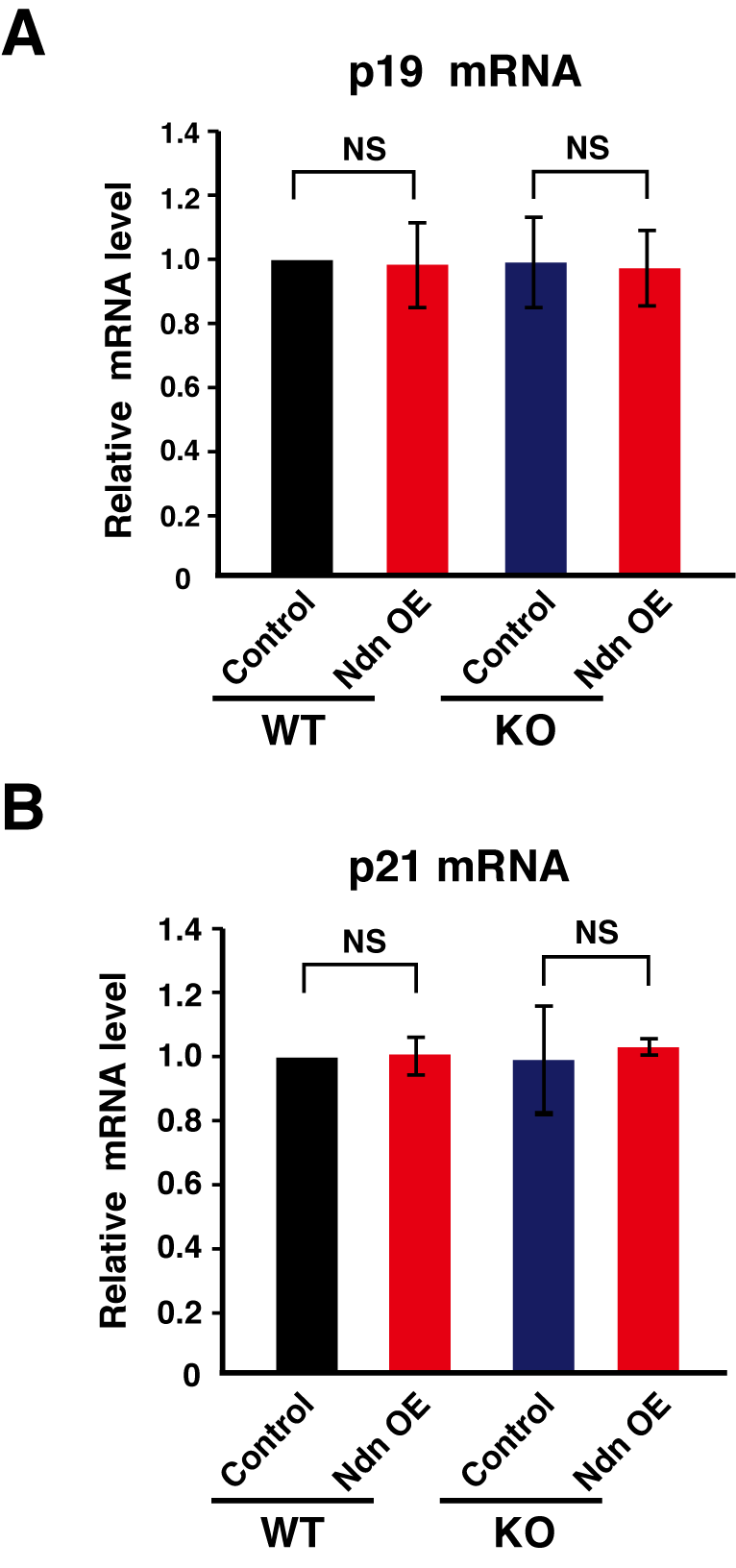

Supplement: Figure S6 — Necdin exerts no modulatory effects on p19 and p21 expression in neocortical NPCs. Neocortical NPCs prepared from wild-type (WT) and necdin-null (KO) mice at E14.5 were infected with lentiviruses for GFP expression (Control) and necdin overexpression (Ndn OE). The p19 (A) and p21 (B) mRNA levels in lentivirus-infected NPCs were analyzed by qRT-PCR. Values represent the mean ± SEM, n = 3. NS, not significant (p>0.05). (TIF) [file pone.0084460.s006.tif]

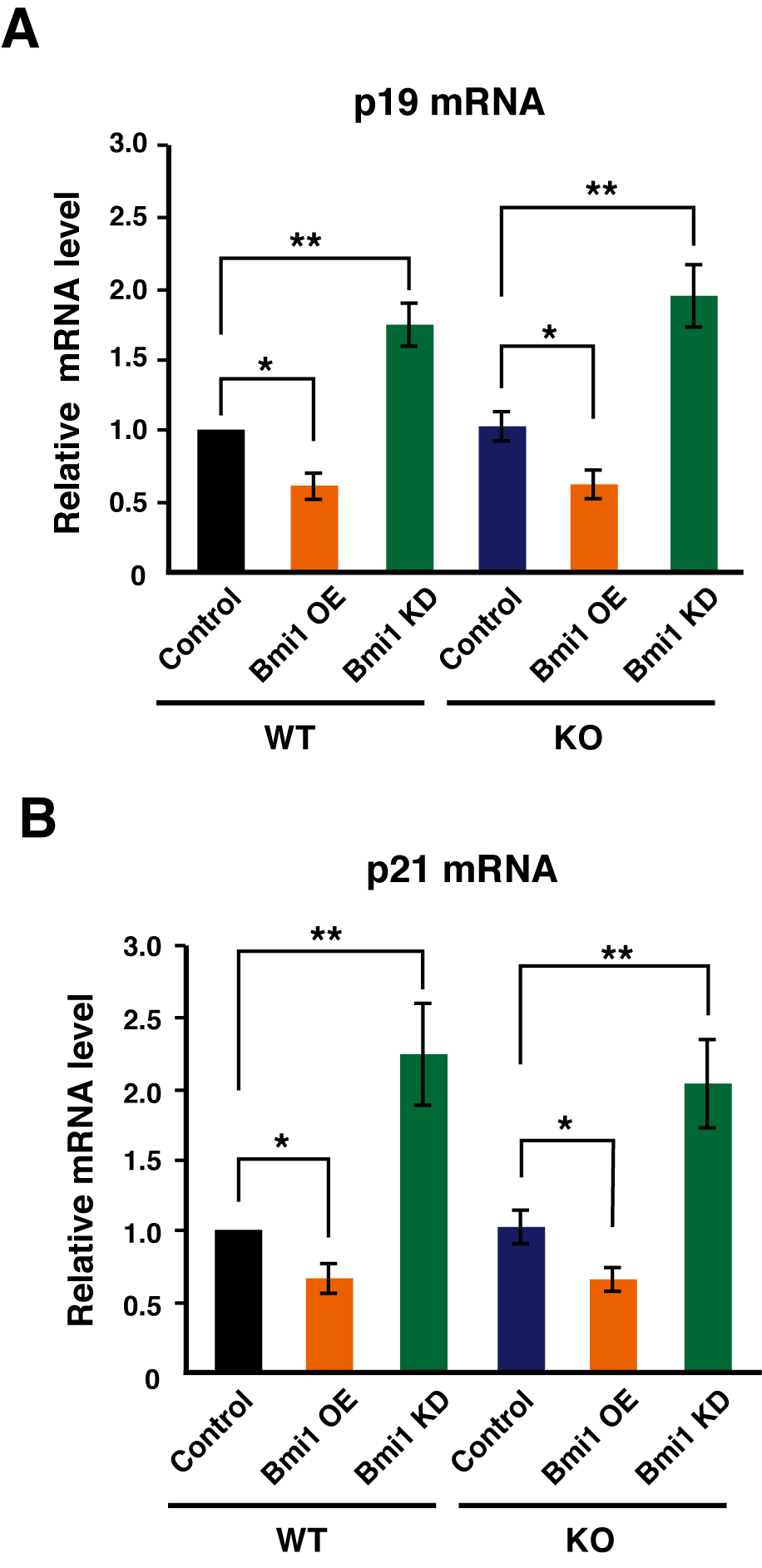

Supplement: Figure S7 — Bmi1 exerts modulatory effects on p19 and p21 expression in neocortical NPCs. Neocortical NPCs prepared from wild-type (WT) and necdin-null (KO) mice at E14.5 were infected with lentiviruses for GFP expression (Control), Bmi1 overexpression (Bmi1 OE), and Bmi1 shRNA (Bmi1 KD). The p19 (A) and p21 (B) mRNA levels in lentivirus-infected NPCs were analyzed by qRT-PCR. Values represent the mean ± SEM, n = 3. *p<0.05, **p<0.01. (TIF) [file pone.0084460.s007.tif]
